# Small Steps to the Big Picture for Health‐Promoting Applications Through the Use of Chickweed (*Stellaria media*): In Vitro, In Silico, and Pharmacological Network Approaches

**DOI:** 10.1002/fsn3.4505

**Published:** 2024-10-03

**Authors:** Gaia Cusumano, Giancarlo Angeles Flores, Mehmet Veysi Cetiz, Umran Kurt, Gunes Ak, Enver Saka, Shaza H. Aly, Omayma A. Eldahshan, Abdel Nasser Singab, Gokhan Zengin, Ismail Senkardes, Maria J. Rodrigues, Luisa Custodio, Carla Emiliani, Paola Angelini

**Affiliations:** ^1^ Department of Chemistry, Biology and Biotechnology University of Perugia Perugia Italy; ^2^ Botanic Garden “Giardino dei Semplici”, Department of Pharmacy “Gabriele d'Annunzio” University Chieti Italy; ^3^ Department of Bioinformatics, Biocenter University of Wurzburg Wurzburg Germany; ^4^ Department of Biology, Science Faculty Selcuk University Konya Turkey; ^5^ Recep Tayyip Erdogan University Department of Chemistry Rize Turkiye; ^6^ Department of Pharmacognosy, Faculty of Pharmacy Badr University in Cairo (BUC) Cairo Egypt; ^7^ Department of Pharmacognosy, Faculty of Pharmacy Ain Shams University Cairo Egypt; ^8^ Center for Drug Discovery Research and Development Ain Shams University Cairo Egypt; ^9^ Department of Pharmaceutical Botany, Pharmacy Faculty Marmara University Istanbul Turkey; ^10^ Centre of Marine Sciences University of Algarve, Campus of Gambelas Faro Portugal

**Keywords:** antioxidant, health‐promoting, HepG2, *Stellaria media*

## Abstract

*Stellaria media L.,* also called chickweed, is widespread in all parts of the world. In the present study, we investigated the biological properties and chemical profiles of different extracts (ethyl acetate, ethanol, ethanol/water, and water) of *S. media*. The chemical profiles were examined using UHPLC/MS/MS technique. Regarding the biological properties, antioxidant properties as well as enzyme‐inhibiting and cytotoxic effects of the extracts were demonstrated by in vitro methods. To obtain further information about the structure‐ability relationship, network pharmacology and molecular docking were also performed. Twelve phenolic compounds were identified in the extracts and most of them were flavonoids (apigenin, kaempferol derivatives, etc.). The water extract showed the best free radical scavenging activity, while the ethanol was the most active in reducing power tests. When inhibiting AChE, the ethyl acetate extract showed the best inhibitory effect. The water extract has a good cytotoxic effect on HepG2 (cell viability: 33.9% at a concentration of 100 g/mL). The analysis, performed using the STRING database, included these 45 cancer‐associated targets. The identified hub genes were TP53, CDKN2A, PTEN, KRAS, and HRAS. In molecular docking analysis, acacetin‐*O*‐hexoside‐*O*‐deoxyhexoside and napigenin‐7‐*O*‐hexoside exhibit remarkable binding energies with proteins. Consequently, *S. media* can be potential raw materials for designing functional formulations in the pharmaceutical, nutraceutical, and cosmeceutical industries.

## Graphical abstract

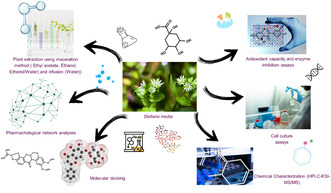

## 1 Introduction

Oxidative stress (OS) caused by the excess of free radicals can lead to cellular and tissue damage, contributing to the onset of chronic and degenerative diseases. This stress is linked to a range of health issues, including cardiovascular diseases, neurodegenerative disorders, cancer, and diabetes (Aly et al. 2021; El‐Nashar et al. 2021; Tan et al. 2020). However, natural antioxidants derived from plants, especially those rich in phenolic compounds, are being recognized for their potential to counteract OS and mitigate its harmful effects (Elgindi et al. 2016; Saracila et al. 2021). Phytotherapy, an evolving and cost‐effective approach, leverages plant‐based secondary metabolites to create new therapeutic agents with minimal side effects (Aly et al. 2022; Khedher et al. 2022; Saber et al. 2024). For example, fruits, vegetables, spices, and herbs are notable sources of antioxidants (Alqethami and Aldhebiani 2021). The growing popularity of herbal medicines can be attributed to their reduced side effects compared to synthetic drugs (El‐Nashar et al. 2022; Kripasana and Xavier 2020). Additionally, the consumption of traditional leafy vegetables, which are abundant in various health‐promoting phytochemicals, is essential for enhancing health (Abdelazim et al. 2024; Kudumela et al. 2024; A. Singab et al. 2015; A. N. B. Singab et al. 2014). Plant‐derived drugs represent an evolving, safe, and cost‐effective strategy and an alternative approach to conventional procedures involving animal cell cultures or microbial fermentation (Elebeedy et al. 2023; Hussiny et al. 2020). Consequently, drugs obtained from natural plant compounds can offer patients more rapid and accessible treatments (Abdelghffar et al. 2022; Eldahshan et al. 2009; Goher et al. 2024; Subramoniam 2014; Veeresham 2012). The genus *Stellaria* L. belongs to the family Caryophyllaceae and comprises about 120 species, according to recent studies, distributed primarily in temperate zones across Asia and Europe (Li, Yang, and Mehri 2021; Salinitro et al. 2020). *S. media* L., also known as “chickweed,” is a small, herbaceous annual plant with oval leaves and small white flowers, widely distributed across Europe, Asia, and North America, often found in gardens and agricultural fields (Ahmad et al. 2022). In Turkey, *S*. *media* is prevalent, particularly in the northern regions, where it contributes to local biodiversity and traditional herbal practices (Güner and Aslan 2012). *S. media* is an edible and medicinal plant, as highlighted by different studies. For example, it is consumed as a leaf vegetable, often raw in salads or lightly cooked (Tutenocakli 2023). In traditional medicine, *S. media* is well‐regarded for helping in weight loss and improving blood lipid profiles. For example, in the Hungarian tradition, *S. media* tea enhances overall metabolism and decreases blood glucose levels, making it a valuable adjunct therapy for diabetic patients (Demján et al. 2022). Additionally, *S. media* is commonly included in daily diets to help manage various conditions such as respiratory issues and skin ailments (such as burns, cuts, and scratches due to its healing effects) (Miere et al. 2023). In various studies, *S. media* has been traditionally used to treat mental tension and inflammations of the renal, digestive, reproductive, and respiratory tracts (Shan et al. 2010). The plant is reported for its antipyretic, anti‐inflammatory (Morita et al. 1996), anticancer (Chon et al. 2009), antifungal, and antibacterial properties (Shinde et al. 2008). It also has diuretic, expectorant, antiasthmatic (Duke 2002), and anxiolytic effects (Arora and Sharma 2012), and is used for rheumatic joint inflammation and wound healing (Arora and Sharma 2014). Regarding the chemical composition, *S. media* contains about 50 bioactive compounds (Oladeji and Oyebamiji 2020), most of which are of phenolic origin such as vanillic acid, p‐hydroxybenzoic acid, ferulic acid, caffeic acid, and chlorogenic acid (Kitanov 1992). The plant also features significant flavonoid and saponin constituents, including apigenin, genistein, vicenin‐2, and gypsogenin (Hodisan and Sancraian 1989). Moreover, it has a total of 16 free amino acids, with 9 being essential amino acids: valine, threonine, isoleucine, leucine, methionine, phenylalanine, histidine, lysine, and arginine (Shan et al. 2010).

In this study, ethyl acetate, ethanol, ethanol/water, and water were utilized as solvents to obtain different extracts from the aerial parts of *S. media*. Each extract was then evaluated for its inhibitory potential against enzymes responsible for non‐infectious human diseases, as well as for its antioxidant effects. UPLC‐ESI‐MS analysis was performed to analyze the composition of each extract. Specifically, the DPPH and ABTS assays were conducted to evaluate the quenching activities, while CUPRAC, FRAP, and phosphomolybdenum assays were utilized to assess reducing power. Additionally, a metal chelating assay was performed to gain more information about the antioxidant potential of the extracts. The targets for evaluating antienzymatic ability included acetylcholinesterase (AChE), butyrylcholinesterase (BChE), tyrosinase, amylase, and glucosidase. Furthermore, the percentage of viability of cell lines, including human embryonic kidney cells (HEK 293), murine macrophages (RAW 264.7), and human hepatocarcinoma cells (HepG2), was calculated to investigate the cytotoxic potential of all the extracts in this study. As an innovative approach, after collecting all chemical and biological activity results, we performed the disease ontology enrichment analysis, protein–protein interaction (PPI) network analysis, and molecular docking to understand the interactions between the components in the UPLC‐ESI‐MS analysis and target proteins and genes. The obtained results can serve as a scientific starting point for further health‐promoting applications with *S. media*.

## 2 Materials and Methods

### 2.1 Plant Collection

In 2022, plant materials were gathered from Maltepe location (Başıbüyük), Istanbul, Turkey. Dr. Ismail Senkardes performed the taxonomic identification, and a voucher specimen was stored in the herbarium of Marmara University (Voucher number: MARE‐22584). The aerial portions were separated, dried in the shade at room temperature, powdered, and then stored away from light.

### 2.2 Plant Extract Preparation

The extraction process involved four solvents: ethyl acetate, ethanol, ethanol/water (70%), and water. Each sample, weighing 10 g, was macerated with 200 mL of ethyl acetate, ethanol, and ethanol/water for a 24‐h period at room temperature. The water extract was created by steeping 10 g of plant material in boiled water for 15 min. Organic solvents were evaporated under reduced pressure, and the water extract underwent freeze‐drying.

### 2.3 Assay for Total Phenolic and Flavonoid Contents

Following the procedures specified by Zengin and Aktumsek (2014), total phenolics and flavonoids were measured. Gallic acid (GA) and rutin (RE) were employed as references in the experiments, with the results presented as gallic acid equivalents (GAE) and rutin equivalents.

### 2.4 UPLC‐ESI‐MS Analysis

A comprehensive analysis was conducted using HPLC‐ESI‐MS/MS to investigate the chemical constitution of various extracts derived from *S. media*.

The samples were introduced into a Shimadzu 8045 (UPLC) system manufactured in Kyoto, Japan, which was linked to a triple quadrupole mass analyzer manufactured by Shimadzu Corporation. The extracts were diluted in HPLC‐quality methanol and filtered through a 0.2 μm polytetrafluoroethylene (PTFE) filter. Chromatography was used to isolate compounds using a Shimpack C18 reversed‐phase column with a particle size of 2.7 μm and dimensions of 2 × 150 mm. Gradient elution was implemented using solvents A (water) and B (acetonitrile) at 0.2 mL/min. Start elution with 10% B concentration for 5 min. The concentration was gradually increased to 30% B for 15 min, then to 70% B for 22 min. The concentration was increased to 80% B from 22 to 30 min. Eventually, the concentration was lowered to 10% B for 35 min. Mass detection was done using negative electrospray ionization (ESI). Set the interface temperature to 300°C and the desolvation temperature to 526°C. Set the cone gas flow rate to 50 L/h and the nebulizing gas flow rate to 3 L/min. Collision‐induced dissociation (CID) (MS/MS) assessments were performed by individually adjusting the collision energy for each peak within a range of 20–50 eV (eV). Mass spectrometry was conducted within the mass range of m/z 100–1200.

The data processing was conducted with the Lab Solutions program. The data collection and analysis procedures were conducted using XcaliburTM2.0.7 software, developed by Thermo Scientific in Karlsruhe, Germany (Aly et al. 2024, 2023; Zengin et al. 2024).

### 2.5 Assays for In Vitro Antioxidant Capacity

As per the procedures outlined in our previous paper (Aly et al. 2022; Grochowski et al. 2017), various antioxidant assays were executed. The outcomes from the DPPH, ABTS radical scavenging, CUPRAC, and FRAP assays were reported in milligrams of Trolox equivalents (TE) per gram of extract. The phosphomolybdenum (PBD) assay indicated the antioxidant potential in millimoles of Trolox equivalents (TE) per gram of extract, while the metal chelating activity (MCA) was expressed in milligrams of disodium edetate equivalents (EDTAE) per gram of extract.

### 2.6 Inhibitory Effects Against Some Key Enzymes

In accordance with the established protocols (Aly et al. 2022; Grochowski et al. 2017), enzyme inhibition experiments were conducted on the samples. The activities inhibiting amylase and glucosidase were expressed in acarbose equivalents (ACAE) per gram of extract, whereas inhibition of acetylcholinesterase (AChE) and butyrylcholinesterase (BChE) was measured in milligrams of galanthamine equivalents (GALAE) per gram of extract. Tyrosinase inhibition was calculated in milligrams of kojic acid equivalents (KAE) per gram of extract.

### 2.7 Cell Culture

Human hepatocarcinoma HepG2, murine macrophages RAW 264.7, and human embryonic HEK 293 cell lines were cultured in Dulbecco's Modified Eagle medium (DMEM) supplemented with fetal bovine serum (10%), L‐glutamine (2 mM, 1%), and penicillin (50 U/mL)/streptomycin (50 μg/mL) (1%), and kept under a humidified atmosphere at 37°C and 5% CO_2_.

### 2.8 Determination of Cellular Viability

Cells were plated in 96‐well plates at 5 × 10^3^ cells/ well (HepG2) and 1 × 10^4^ cells/ well (RAW 264.7 and HEK 293). After an overnight incubation, cells were treated with the extracts at the concentration of 100 μg/mL for 72 h. Cells incubated with DMSO at 0.5% were used as control. The cellular viability was determined by the MTT (3‐(4,5‐dimethylthiazol‐2‐yl)‐2,5‐diphenyltetrazolium bromide) test, as described formerly (Rodrigues et al. 2016). The percentage of cellular viability was calculated relative to the control (DMSO 0.5%).

### 2.9 Disease Ontology Enrichment Analysis

A comprehensive Disease Ontology (DOSE) enrichment analysis was conducted using the DOSE package (version 3.30.1) in R. This methodology is based on the protocols established by Yu et al. (2014) and other relevant literature in the fields of bioinformatics and systems biology. The genes associated with the molecules were sourced from TMCSD, PubChem, and SwissTarget. The DOSE package integrates semantic similarity measures and enrichment analysis to explore the associations between genes and diseases. The enrichment analysis identifies overrepresented disease terms linked to a set of genes, thereby providing insights into potential disease‐related pathways. DOSE employs a hypergeometric distribution model to calculate *p* values, which identify statistically significant disease terms. Additionally, semantic similarity scores are calculated to assess the degree of relatedness between the identified diseases. This ensures that the gene sets associated with molecules are accurately analyzed within the context of disease ontology (Yu et al. 2014).

### 2.10 Screening of Potential Targets

The identification of therapeutic targets represents a pivotal stage in the development of novel pharmaceutical agents within the field of medical research. In this context, the Comparative Toxicogenomics Database (CTD), PubChem, GeneCards, and DisGeNET databases are of significant value as tools for identifying potential drug targets in the fight against cancer. A search for the term “renal cell carcinoma” in the four aforementioned databases revealed a number of related genes. The genes associated with the compounds were then identified using the online databases TMCSD, PubChem, and SwissTarget. Common targets were identified using the Venny V2.1.0 online tool, with the aim of identifying targets that were relevant to the effects of the compounds on renal cell carcinoma (Yagi et al. 2024).

### 2.11 Protein–Protein Interaction (PPI) Network Analysis

A protein–protein interaction (PPI) network was constructed using the STRING V12.0 database in order to investigate the renal cell carcinoma properties of *S. media*. This analysis focused on the functional interactions between proteins. A network confidence value of ≥ 0.4 was employed to identify potential targets, with “*Homo sapiens*” specified as the species of interest in the Cytoscape software, version 3.10.2 (Yagi et al. 2024).

### 2.12 Molecular Docking

The proteins and enzymes utilized in this study were sourced from the Protein Data Bank (PDB) (for further details, please refer to Table S1). Subsequently, the co‐crystallized ligands, cofactors, and water molecules were removed using BIOVIA Discovery Studio Visualizer V4.5. The ligands were obtained from PubChem, while those not present in the database were created using ChemDraw V23.1.1 and subsequently optimized with OpenBabel V3.1.1. The ligand energy minimization process was conducted using Avogadro V0.8.0 with the MMFF94 force field. The preparation of protein and enzyme structures was conducted using MGL Tools software, version 1.5.6. The active sites of the proteins were identified using POCASA V1.1 and relevant literature (see Table S1) (Hetmann et al. 2023; Duran et al. 2024). To validate the docking results, a re‐docking process was conducted. The ligand was re‐docked with the protein, and the root mean square deviation (RMSD) values were calculated to assess the accuracy of the docking process (Castro‐Alvarez, Costa, and Vilarrasa 2017). The root mean square deviation (RMSD) was calculated using the following formula to measure the average deviation between the positions of atoms in the reference and target structures:

$$\text{RMSD}=\sqrt{\frac{1}{N}\sum_{i=1}^{N}{\left(r_{i}^{\mathrm{ref}}-r_{i}^{\text{target}}\right)}^{2}}$$

Molecular docking was conducted using AutoDock Vina V1.1.2, with grid boxes defined in accordance with the methodology outlined by Trott and Olson (2010).

## 3 Results and Discussion

### 3.1 Total Phenolic and Flavonoid Content

Studies have shown that the total phenolic and flavonoid contents in various plant extracts significantly correlate with their antioxidant activities, emphasizing the importance of these compounds in natural antioxidants (Syed Salleh et al. 2021). The Folin–Ciocalteu assay is a colorimetric test widely used in the evaluation of the content of phenols and flavonoids from natural products (Dominguez‐López, Pérez, and Lamuela‐Raventós 2023; Narvarte et al. 2023). In this work, different extracts derived from the aerial parts of *S. media* were tested in the Folin–Ciocalteu assay to determine the total phenolic and flavonoid content. In Table 1, it can be seen that the highest level of phenolics was found in the ethanol and ethyl acetate extracts, with values of 22.76 ± 0.07 and 22.71 ± 0.82 mg GAE/g, respectively. Other extracts showed lower values of TPC (14.22 ± 0.04 mg GAE/g for water and 14.10 ± 0.08 mg GAE/g for ethanol/water). Regarding the total flavonoid content, the best result was obtained with ethanol/water, with a value of 6.75 ± 0.31 mg RE/g. Then the TFC order was water > ethanol > ethyl acetate, with values of 5.75 ± 0.14, 2.57 ± 0.43, and 1.13 ± 0.08 mg RE/g, respectively. In the study by Rakhimzhanova, Kılınçarslan, and Mammadov (2018), the ethanolic extract of dried *S. media* showed the highest total phenolic content with a value of 21.43 ± 0.12 mg GAE/g. These findings agree with our results. However, the aerial part extract of *S. media* tested in our work showed lower values of total flavonoid content. This discrepancy may be attributed to the fact that the extracts used in this study, prepared exclusively from the aerial parts of the plant, demonstrated lower values of total flavonoid compounds compared to the study by Rakhimzhanova, Kılınçarslan, and Mammadov (2018), which employed the whole plant for extract preparation.

**TABLE 1:** Total phenolic and flavonoid contents in the tested extracts.

Extracts	TPC (mg GAE/g)	TFC (mg RE/g)
Ethyl acetate	22.71 ± 0.82^a^	1.13 ± 0.08^d^
Ethanol	22.76 ± 0.07^a^	2.57 ± 0.43^c^
Ethanol/water	14.10 ± 0.08^b^	6.75 ± 0.31^a^
Water	14.22 ± 0.04^b^	5.75 ± 0.14^b^

*Note:* Values are reported as mean ± SD of three parallel measurements. Different letters (a,b, c and d) indicate significant differences between the extracts (*p* < 0.05).

Abbreviations: GAE, Gallic acid equivalents; RE, Rutin equivalents.

### 3.2 UPLC‐ESI‐MS/MS Profiling of the Different Extracts of *Stellaria media*

**FIGURE 1:**
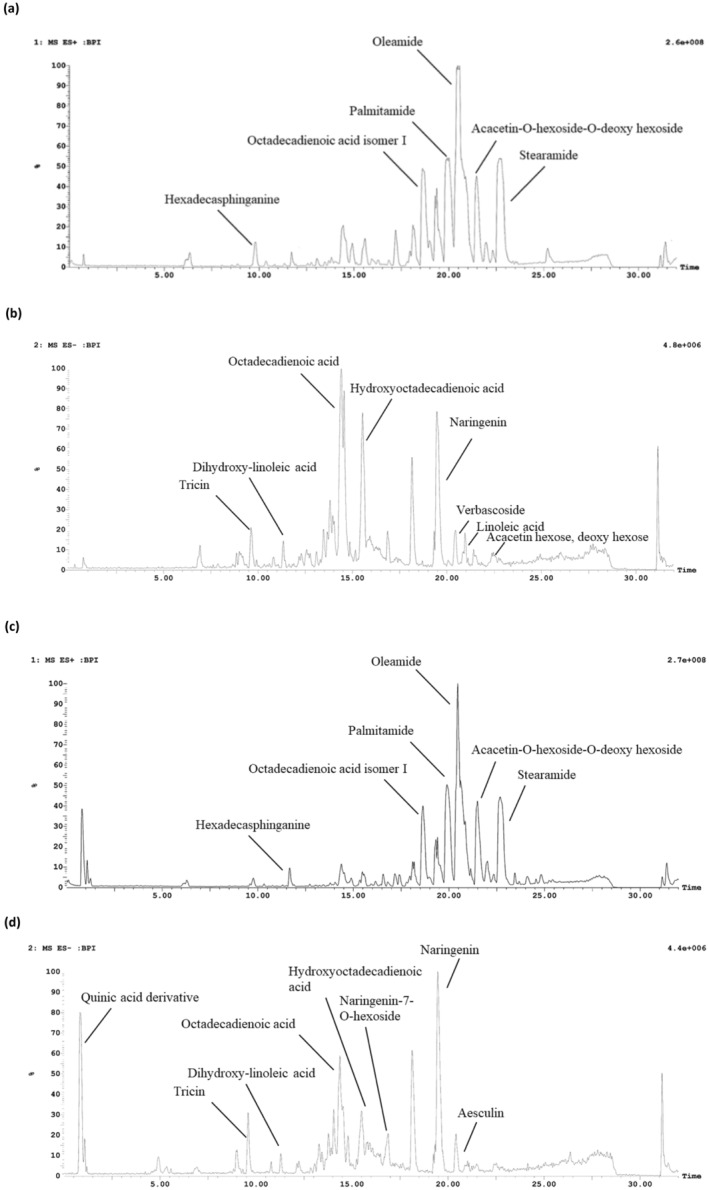
The HPLC chromatograms of *S. media*: (a) ethyl acetate extract positive mode, (b) ethyl acetate extract negative mode, (c) ethanol extract positive mode, (d) ethanol extract negative mode.

**FIGURE 2:**
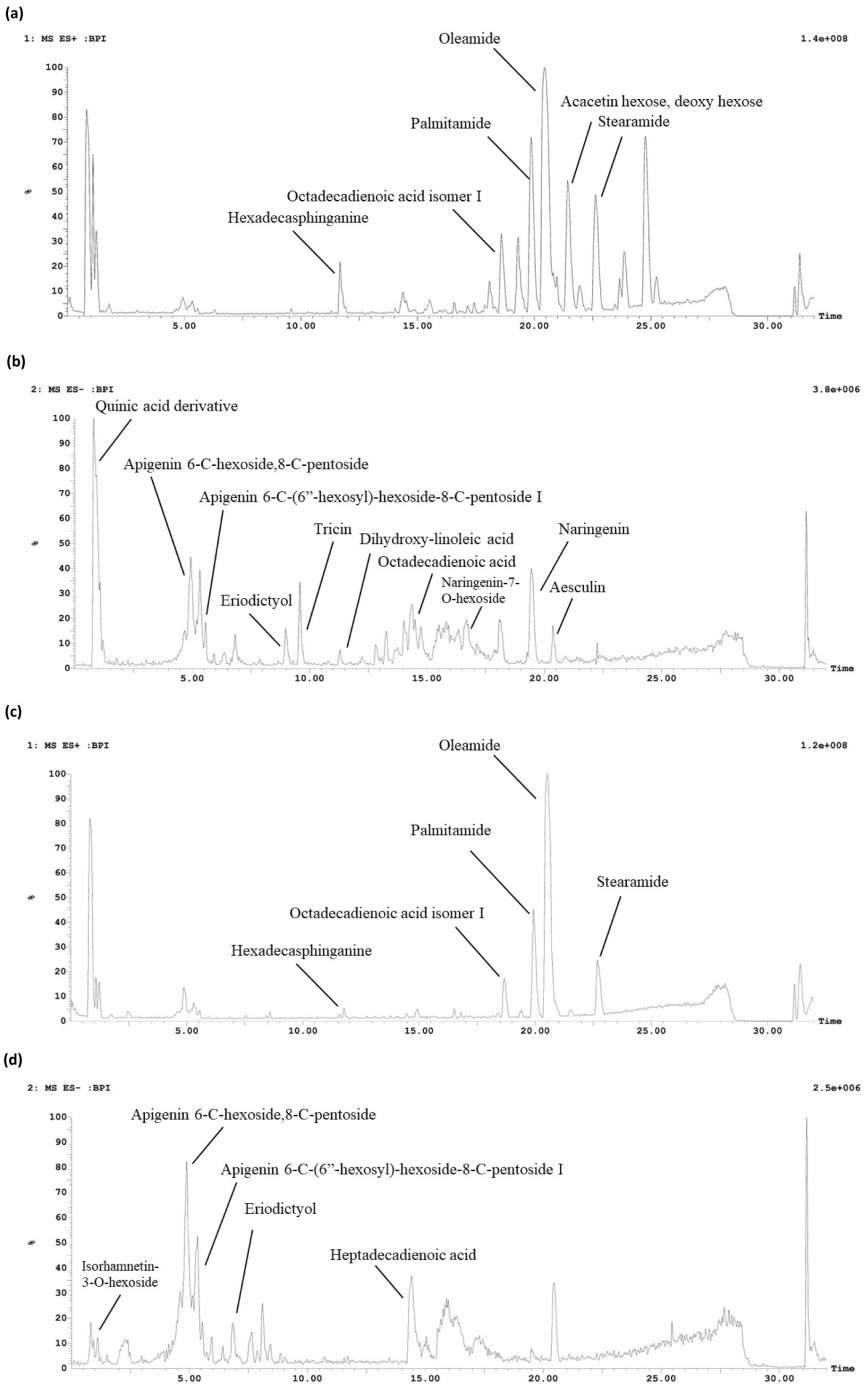
The HPLC chromatograms of *S. media*: (a) ethanol/water extract positive mode, (b) ethanol/water extract negative mode, (c) water extract positive mode, (d) water extract negative mode.

An investigation of the variability in the chemical composition of the *S. media* (Caryophyllaceae) different extracts of ethyl acetate, ethanol, ethanol/water, and water was performed using LC–MS/MS analysis. The total ion chromatogram revealed the presence of 24 distinct chromatographic peaks (Figures 1 and 2). There are a total of 12 phenolic compounds, consisting of nine flavonoids, two phenolic acids, and one coumarin. In addition, seven fatty acids, four fatty amides, and one sphingolipid were successfully identified by analyzing their molecular ion peaks, MS^2^ data, and comparing them with existing literature (Table 2).

**TABLE 2:** Metabolites identified tentatively in the ethyl acetate (EtOAc), ethanol, ethanol/water, and water extracts of *Stellaria media* from Turkey using UPLC‐ESI‐MS/MS analysis.

Peak no.	*t*_R_	[M‐H]^−^	[M + H]^+^	MS^2^	Tentatively identified compounds	Phytochemical class	Relative area %				Ref.
							*Stellaria media*				
							EtOAc	Ethanol	Ethanol/water	Water	
1	0.79	377.17	—	191, 93	Quinic acid derivative	Quinic acid derivative	—	12.51	23.95	—	(Nilofar et al. 2024)
2	1.07	477.29	—	477, 315, 314, 285	Isorhamnetin‐3‐O‐hexoside	Flavonoid	—	—	—	1.07	(AbouZeid et al. 2022)
3	4.93	563.28	—	443,353, 269	Apigenin 6‐C‐hexoside 8‐C‐pentoside	Flavonoid	—	—	16.87	42.74	(Katanić Stanković et al. 2023)
4	5.57	725.38	727.37	443, 353, 365, 383, 384, 425	Apigenin 6‐C‐(6″‐hexosyl)‐hexoside‐8‐C‐pentoside I	Flavonoid	—	—	1.52	1.95	(Katanić Stanković et al. 2023)
5	6.41	739.35	—	285, 241, 254, 227	Kampferol‐O‐hexoside‐O‐dideoxyhexoside	Flavonoid	—	—	—	0.51	(Abdelghffar et al. 2021)
6	6.84	287.19	—	151, 135, 107	Eriodictyol	Flavonoid	—	—	1.36	2.56	(Katanić Stanković et al. 2023)
7	9.62	329.32	—	249, 175, 229, 211, 171	Tricin	Flavonoid	2.50	3.51	4.51	—	(Raslan et al. 2021)
8	11.34	311.24	—	293, 267, 171, 153	Dihydroxy‐linoleic acid	Fatty acid	1.13	0.91	0.69	—	(Ayoub et al. 2021)
9	11.71	—	274.30	106, 256, 274	Hexadecasphinganine	Sphingolipid	0.49	1.00	1.30	0.63	(AbouZeid et al. 2022)
10	14.41	265.22		—	Heptadecadienoic acid	Fatty acid	—	—	—	11.11	(AbouZeid et al. 2022)
11	14.43	293.27	—	275, 231, 183, 211, 121	Octadecadienoic acid	Fatty acid	15.74	9.98	4.31	—	(Zengin et al. 2022)
12	14.57	293.26	—	275, 231, 183, 211, 121	Octadecadienoic acid	Fatty acid	9.46	3.34	1.94	—	(Zengin et al. 2022)
13	15.58	295.29	—	277, 195, 183, 171	Hydroxyoctadecadienoic acid	Fatty acid	11.79	5.89	—	—	(Zengin et al. 2022)
14	16.68	433.34	—	271	Naringenin‐7‐O‐hexoside	Flavonoid	—	0.25	2.79	—	(Rodrigues et al. 2016)
15	18.62	—	280.33	109, 133	Octadecadienoic acid isomer I	Fatty acid	4.14	2.39	3.00	3.50	(AbouZeid et al. 2022)
16	19.47	271.30	—	225, 241, 151, 119	Naringenin	Flavonoid	7.35	19.85	9.21	0.71	(Abdelghffar et al. 2021)
17	19.89	—	256.34	102, 116, 57	Palmitamide	Fatty acid amide	3.71	9.75	3.25	10.75	(Zengin et al. 2022)
18	20.01	—	256.35	102, 116, 57	Palmitamide	Fatty acid amide	4.08	4.13	6.76	—	(Zengin et al. 2022)
19	20.35	—	282.57	97,83, 57, 69, 149, 163	Oleamide	Fatty acid amide	12.36	13.37	8.85	23.18	(Zengin et al. 2022)
20	20.45	623.4166	—	315, 461, 462	Verbascoside	Phenolic acid	2.31	—	—	—	(Katanić Stanković et al. 2023)
21	20.46	339.34	—	177, 163	Aesculin	Coumarin	—	2.74	2.00	7.02	(Hamdan et al. 2022)
22	20.94	279.31	—	279	Linoleic acid	Fatty acid	1.22	0.20	—	—	(Fahmy et al. 2024)
23	21.40	591.41	593.43	284, 242	Acacetin‐O‐hexoside‐O‐deoxy hexoside	Flavonoid	5.63	5.65	6.73	—	(El‐Nashar et al. 2023)
24	22.70	—	284.40 567.70 [2 M + 1]^+^	88, 102, 57	Stearamide	Fatty acid amide	10.74	12.52	4.96	5.73	(Castillo‐Peinado et al. 2019)

The findings demonstrated that flavonoids were particularly concentrated in the ethyl acetate, ethanol, and ethanol/water extracts, but fatty acids and fatty acid amides were mostly present in the ethyl acetate extract. Furthermore, water was an effective solvent for extracting phenolic acids and flavonoid glycosides. Flavonoids are recognized as the main group of plant compounds in *S. media* and are accountable for its numerous biological properties (Aleem et al. 2023; Oladeji and Oyebamiji 2020).

Apigenin and apigenin C‐glycosides were previously identified in *S. nemorum* and *S. holostea* (Ancheeva et al. 2015). Peaks **3** and **4** show a parent ion peak at *m/z* 563 and 725 [M‐H]^−^, respectively. A daughter ion peak at [M‐H–120]^−^ was also observed in the product ion spectra and was a characteristic production of C‐glycosides (Sun et al. 2009). Peaks **3** and **4** were identified as apigenin 6‐C‐hexoside 8‐C‐pentoside and apigenin 6‐C‐(6″‐hexosyl)‐hexoside‐8‐C‐pentoside I.

Moreover, flavonoid *O*‐glycosides, isorhamnetin‐3‐O‐hexoside (**2**) and kampferol‐O‐hexoside‐O‐dideoxyhexoside (**5**) with their deprotonated molecular ion at *m/z* 477 and 739, respectively, were identified in the water extract. Another two flavonoid glycosides were characterized as naringenin‐7‐O‐hexoside (**14**) and acacetin‐O‐hexoside‐O‐deoxy hexoside (**23**) with a parent ion peak at *m/z* 433 and 591 [M‐H]^−^, respectively.

Flavonoid aglycones are among the most abundant secondary metabolites in the genus *Stellaria* as previously investigated in the *S. holostea* methanol extract (Katanić Stanković et al. 2023). Concerning the detected flavonoid aglycones in *S. media*, eriodictyol (**6**) and tricin (**7**) were characterized based on their parent ion peaks at *m/z* 287 and 329 [M‐H]^−^ and their characteristic ions at *m/z* 151, 135 and 249, 175, respectively. The tentatively identified compound in Peak **16** is naringenin, which has a parent ion peak at m/z 271[M‐H]^−^. It is found in all four extracts, but its quantity varies. It is particularly abundant in ethanol and ethanol/water extracts.

Phenolic acid namely the derivative of quinic acid (**1**), was detected at *m/z* 377. It was exclusively extracted using ethanol/water and is a significant constituent of the water extract. Also, verbascoside (**20**) was detected at *m/z* 623, it is a derivative of caffeic acid and was previously identified in *S. holostea* methanol extract (Katanić Stanković et al. 2023). One coumarin was identified as aesculin (**21**) with a molecular ion peak ate *m/z* 339 in the four extracts and it had the highest abundance in the water extract.

In addition, the ethyl acetate and ethanol extracts of *S. media* were shown to contain significant amounts of fatty acids, specifically dihydroxy‐linoleic acid, heptadecadienoic acid, octadecadienoic acid, hydroxy octadecadienoic acid, octadecadienoic acid isomer I, and linoleic acid. Furthermore, fatty acid amides have a higher retention time in the chromatogram, specifically between 19.89 min and 22.70 min. These compounds are identified as palmitamide, oleamide, and stearamide. They displayed the distinctive molecular ion fragment of an acyl chain, which was identified at *m/z* 57 (Divito et al. 2012).

### 3.3 Antioxidant Effects

The assessment of the antioxidant activity of plant extracts is one of the fundamental steps in the process that transforms natural resources into pharmaceutical products. For this reason, in this study, we investigated the scavenging ability with DPPH and ABTS assays, the reducing power with CUPRAC, FRAP, and Phosphomolybdenum assays, and the metal chelating capacity. Results are reported in Table 3. The best scavenging ability in the DPPH assay was observed for the water extract with a value of 13.04 ± 0.36 mg TE/g, followed by ethanol/water with a value of 10.10 ± 0.96 mg TE/g, and ethanol with 2.62 ± 0.30 mg TE/g. No DPPH scavenging ability was observed for the ethyl acetate extract. Regarding the ABTS assay, water was the best extract with a value of 59.98 ± 0.57 mg TE/g, followed by ethanol/water (39.13 ± 1.66 mg TE/g), ethanol (26.67 ± 1.19 mg TE/g), and ethyl acetate (12.94 ± 0.88 mg TE/g). In the CUPRAC, FRAP, and Phosphomolybdenum assays, the transformation of Cu^2+^ to Cu^+^, Fe^3+^ to Fe^2+^, and Mo(VI) to Mo(V), respectively, is evaluated. In the CUPRAC assay, the extract that showed the highest reducing power was ethanol with a value of 61.60 ± 1.00 mg TE/g, followed by ethyl acetate with 55.82 ± 1.12 mg TE/g, ethanol/water with 36.82 ± 1.01 mg TE/g, and water (24.28 ± 0.02 mg TE/g). In the FRAP assay, ethanol was the best, with 27.55 ± 0.24 mg TE/g, followed by water with 26.46 ± 0.24 mg TE/g, ethanol/water with 25.93 ± 0.27 mg TE/g, and ethyl acetate with a value of 25.11 ± 1.17 mg TE/g. In the Phosphomolybdenum assay (PBD), the best ability was observed for ethyl acetate (2.05 ± 0.07 mmol TE/g), followed by ethanol (1.97 ± 0.06 mmol TE/g). Ethanol/water and water showed the weakest ability, with 0.82 ± 0.04 mmol TE/g and 0.22 ± 0.01 mmol TE/g, respectively. The chelation of transition metals is a very important antioxidant mechanism to stop the production of hydroxyl radicals in the Fenton reaction. Among the tested extracts in this study, the ethyl acetate extract revealed the best chelating ability, with a value of 25.60 ± 0.61 mg EDTAE/g. For water and ethanol/water extracts, similar results were observed (17.96 ± 0.24 mg EDTAE/g and 17.21 ± 0.84 mg EDTAE/g, respectively). In other works, the antioxidant capacity of *S. media* had already been evaluated using DPPH, and the results were expressed as scavenging percentage and IC50, which are not comparable to our results. In the study by Rakhimzhanova, Kılınçarslan, and Mammadov (2018), the ethanol extracts with an IC50 value of 49.72 ± 0.57 showed higher radical scavenging activity compared to water extracts. Aleem et al. (2023) found that the extract of *S. media* exhibited a high‐level antioxidant capacity with a scavenging percentage of 76% for DPPH, as stated by Oladeji and Oyebamiji (2020).

**TABLE 3:** Antioxidant properties of the tested extracts.

Extracts	DPPH (mg TE/g)	ABTS (mg TE/g)	CUPRAC (mg TE/g)	FRAP (mg TE/g)	Chelating (mg EDTAE/g)	PBD (mmol TE/g)
Ethyl acetate	na	12.94 ± 0.88^d^	55.82 ± 1.12^b^	25.11 ± 1.17^c^	25.60 ± 0.61^a^	2.05 ± 0.07^a^
Ethanol	2.62 ± 0.30^c^	26.67 ± 1.19^c^	61.60 ± 1.00^a^	27.55 ± 0.24^a^	9.18 ± 0.46^c^	1.97 ± 0.06^a^
Ethanol/water	10.10 ± 0.96^b^	39.13 ± 1.66^b^	36.82 ± 1.01^c^	25.93 ± 0.27^b^	17.21 ± 0.84^b^	0.82 ± 0.04^b^
Water	13.04 ± 0.36^a^	59.98 ± 0.57^a^	24.28 ± 0.02^d^	26.46 ± 0.24^b^	17.96 ± 0.24^b^	0.22 ± 0.01^c^

*Note:* Values are reported as mean ± SD of three parallel measurements. Different letters (a, b, c and d) indicate significant differences between the extracts (*p* < 0.05).

Abbreviations: EDTAE, EDTA equivalent; MCA, metal chelating Activity; na, not active; PBD, phosphomolybdenum; TE, Trolox Equivalent.

### 3.4 Enzyme Inhibitory Effects

Inhibiting enzymes involved in Alzheimer's disease, hyperpigmentation, and type‐2 diabetes, natural enzyme inhibitors play an important role in the treatment of non‐infectious diseases. In this regard, extracts from the aerial parts of *S. media* were tested against AChE, BChE, Tyrosinase, Amylase, and Glucosidase. The results are reported in Table 4. In AChE inhibition, ethyl acetate showed the best results with 2.44 ± 0.04 mg GALAE/g, followed by ethanol with 2.25 ± 0.10 mg GALAE/g, ethanol/water with 1.87 ± 0.07 mg GALAE/g, and water (0.30 ± 0.05 mg GALAE/g). Also, in the BChE inhibition assay, ethyl acetate extract was the strongest, with a value of 3.98 ± 0.39 mg GALAE/g. Ethanol showed similar results (3.95 ± 0.40 mg GALAE/g). Ethanol/water, compared to the other extracts tested in this study, exhibited a weak ability to inhibit BChE with a value of 0.45 ± 0.06 mg GALAE/g, and water was not active. Regarding tyrosinase inhibition, the most potent ability was detected for ethanol and ethanol/water extracts, with values of 51.99 ± 0.50 mg KAE/g and 48.02 ± 1.17 mg KAE/g, respectively. In this case, water showed 12.77 ± 0.47 mg KAE/g and ethyl acetate was not active. Ethyl acetate extract was the best to inhibit amylase (0.79 ± 0.03 mmol ACAE/g), followed by ethanol extract with a value of 0.76 ± 0.03 mmol ACAE/g, ethanol/water (0.48 ± 0.01 mmol ACAE/g), and water (0.09 ± 0.01 mmol ACAE/g). The most active against glucosidase was ethanol (1.19 ± 0.04 mmol ACAE/g), followed by ethanol/water with a value of 0.91 ± 0.12 mmol ACAE/g, and water (0.36 ± 0.01 mmol ACAE/g). This time, ethyl acetate extract was the weakest (0.34 ± 0.01 mmol ACAE/g). There are not many studies in the literature regarding the enzymatic inhibition capacity of *S. media* extracts. However, in the study by Khan, Ahmad, and Ahmed (2019), the extract of *S. media* showed promising inhibitory activities on the enzymes α‐amylase and β‐glucosidase, suggesting its potential use as a natural treatment for controlling postprandial glucose levels in diabetic patients.

**TABLE 4:** Enzyme inhibitory properties of the tested extracts.

Solvents	AChE (mg GALAE/g)	BChE (mg GALAE/g)	Tyrosinase (mg KAE/g)	Amylase (mmol ACAE/g)	Glucosidase (mmol ACAE/g)
Ethyl acetate	2.44 ± 0.04^a^	3.98 ± 0.39^a^	na	0.79 ± 0.03^a^	0.34 ± 0.01^c^
Ethanol	2.25 ± 0.10^b^	3.95 ± 0.40^a^	51.99 ± 0.50^a^	0.76 ± 0.03^a^	1.19 ± 0.04^a^
Ethanol/water	1.87 ± 0.07^c^	0.45 ± 0.06^b^	48.02 ± 1.17^b^	0.48 ± 0.01^b^	0.91 ± 0.12^b^
Water	0.30 ± 0.05d	na	12.77 ± 0.47^c^	0.09 ± 0.01^c^	0.36 ± 0.01^c^

*Note:* Values are reported as mean ± SD of three parallel measurements. Different letters (a, b, c and d) indicate significant differences between the extracts (*p* < 0.05).

Abbreviations: ACAE, acarbose equivalent; GALAE, galantamine equivalent; KAE, kojic acid equivalent; na, not active.

### 3.5 Cytotoxic Effects

Extracts from the aerial parts of *S. media* were utilized to assess cytotoxicity experiments against 3 different cell lines, including HEK 293, RAW 264.7, and HepG2, at a single concentration of 100 mg/mL. The control contained 0.5% DMSO and the results, reported in Table 5, were expressed as a percentage of cellular viability. Ethyl acetate and water extract exhibited the best cytotoxicity effect against the HEK 293 cell line, with a viability of 17.8 ± 2.5% and 19.9 ± 3.7%, respectively; while for ethanol, the result was 27.3 ± 4.1% and for ethanol/water 33.0 ± 3.9%. Against the RAW 264.7 cell line, the best cytotoxic effect was observed for the water extract with a moderate cytotoxic effect (45.1 ± 1.0% of cellular viability) and the lower cytotoxicity was noted for the ethyl acetate extract (74.5 ± 3.5%). As reported from this study, cytotoxicity experiment against the HepG2 cell line results, water exhibited the least cellular viability, with a value of 33.9 ± 0.7%, followed by ethyl acetate (50.1 ± 2.8%), ethanol/water (51.2 ± 2.2%), and ethanol (55.8 ± 3.3%). In our study, the highest sensitivity to *S. media* aerial parts extracts was observed in the HEK 293 cell line, with values ranging from 17.8 ± 2.5% to 33.0 ± 3.9%. This suggests the potential of *S. media* extracts to be therapeutically approached in targeting cancer cells. On the other hand, the least sensitivity was observed for HepG2, with values ranging from 45.1 ± 1.0% to 74.5 ± 3.5%, making the extracts potentially appropriate for applications that need less harmful effects. Regarding the cytotoxic effect of *S. media* extracts, the study by Ma et al. (2012) highlighted those concentrations below 30 μg/mL (including the concentration of 100 μg/mL tested in this study) do not show cytotoxic effects on HepG2 cells. These results, however, cannot be compared with ours since the extracts were not prepared in the same way.

**TABLE 5:** Cellular viability (%) of the tested extracts on mammalian RAW, HepG2, and S17 cell lines, applied at 100 μg/mL.

Cell lines/samples	0.5% DMSO	Ethyl acetate	Ethanol	Ethanol/water	Water
HEK 293	95.1 ± 3.1	17.8 ± 2.5	27.3 ± 4.1	33.0 ± 3.9	19.9 ± 3.7
RAW 264.7	89.2 ± 3.5	74.5 ± 3.5	70.2 ± 2.2	71.0 ± 4.6	45.1 ± 1.0
HepG2	99.5 ± 3.4	50.1 ± 2.8	55.8 ± 3.3	51.2 ± 2.2	33.9 ± 0.7

*Note:* Extracts were tested at 100 μg/mL, and results are expressed as a percentage of cellular viability (%) relatively to the control containing 0.5% DMSO. Values represent the mean ± standard error of the mean (SEM).

### 3.6 Disease Ontology Enrichment Analysis

**FIGURE 3:**
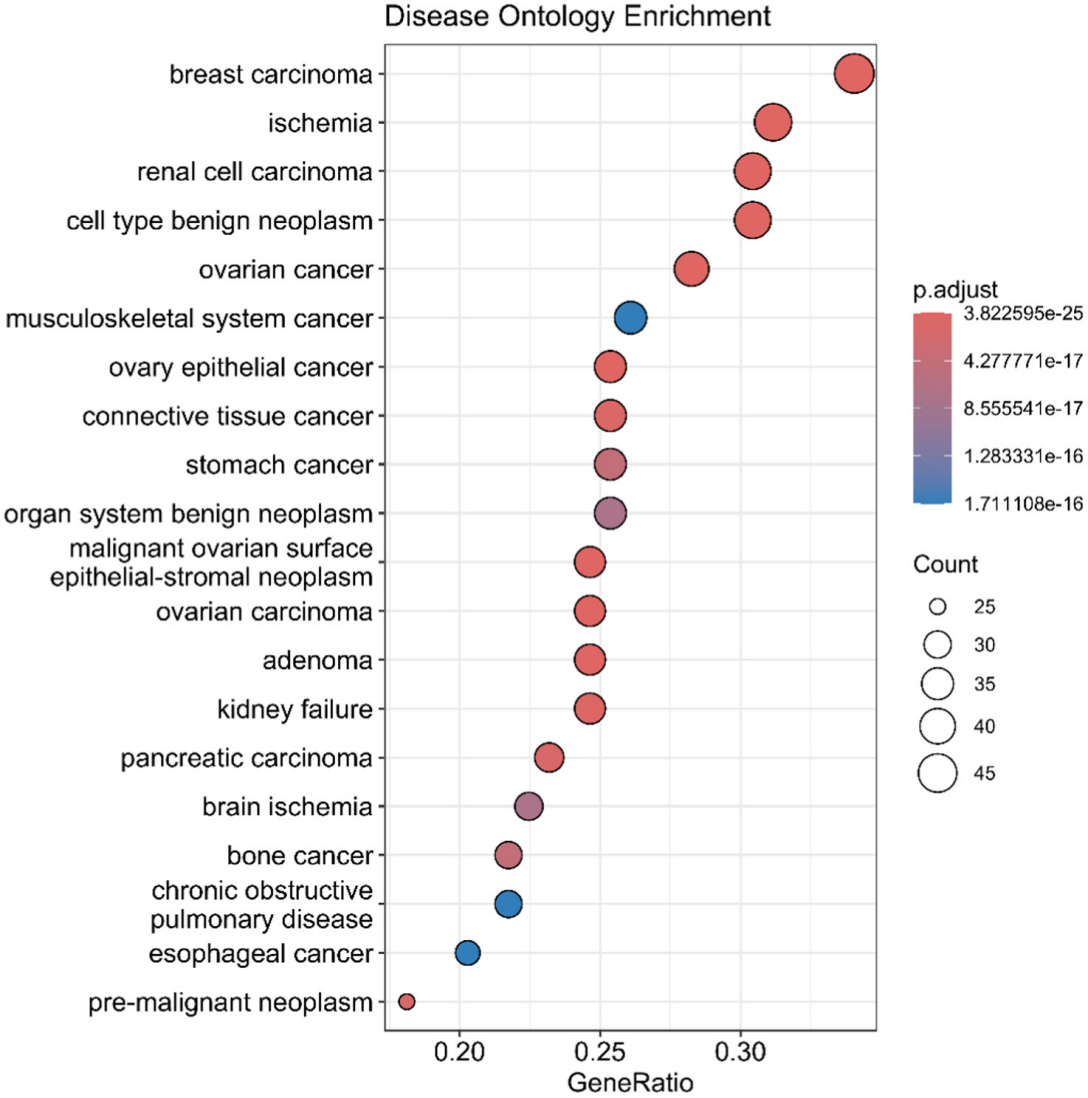
Gene‐disease associations related to *Stellaria media*: DOSE analysis.

The Disease Ontology enrichment analysis (DOSE), conducted in the present study, revealed significant associations between the gene sets of interest and a number of diseases. These findings offer vital insights into the potential biological functions and disease implications of the genes under investigation. The enrichment analysis identified significant associations with multiple types of cancer, including renal cell carcinoma (Figure 3). These findings suggest that the genes under investigation may play a role in oncogenic processes, underscoring their significance in cancer biology.

Furthermore, the identification of diseases such as pre‐malignant neoplasm, brain ischemia, and kidney failure indicate the potential involvement of these genes in these conditions. In view of the pronounced enrichment for cancers, including renal cell carcinoma, these diseases were the subject of particular attention in the subsequent docking studies. The prominence of renal cell carcinoma in the enrichment analysis afforded a robust context for examining the interactions of our compounds with key cancer‐related proteins. Furthermore, ischemia, which is characterized by intricate molecular mechanisms, was selected for investigation into the potential therapeutic applications of the genes under study. These selections were made in order to capitalize on the robust disease associations revealed by the enrichment analysis and to concentrate on conditions with high clinical relevance and unmet medical needs.

### 3.7 *Stellaria media* of Active Compounds: Targeting Cancer

**FIGURE 4:**
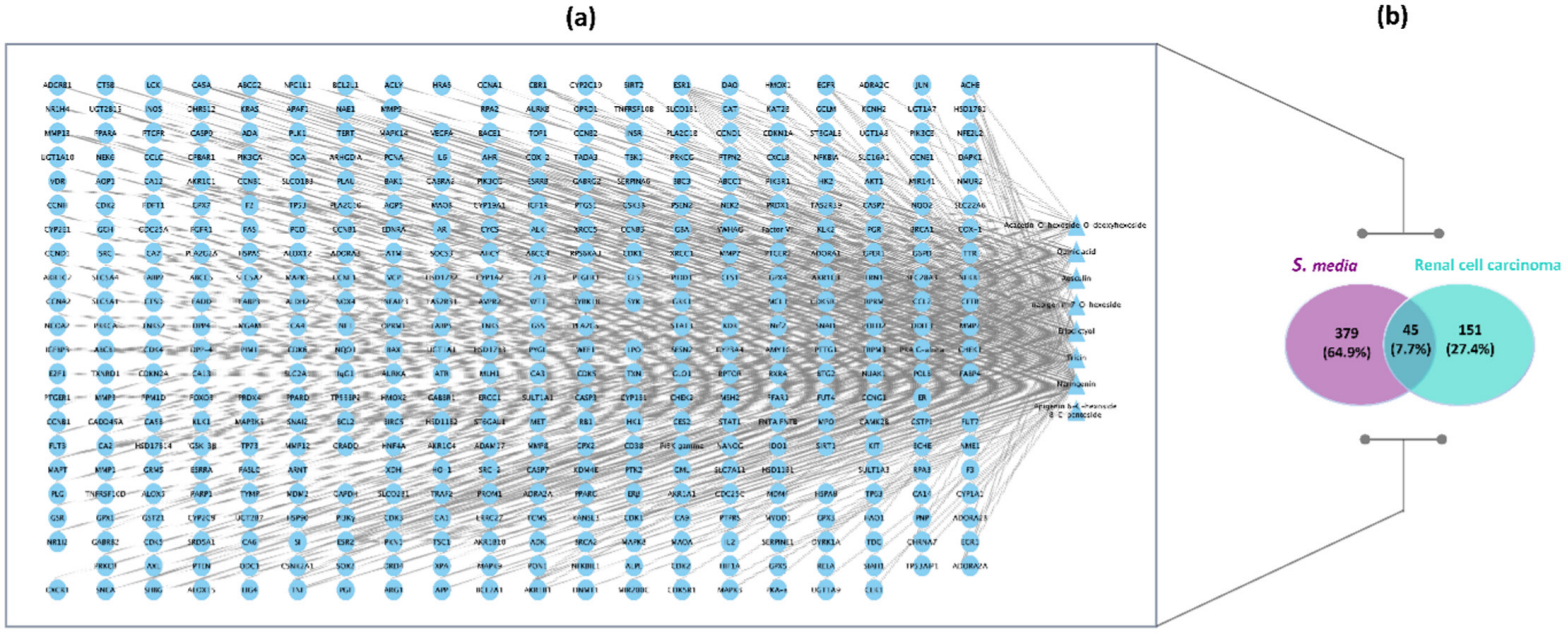
Target analysis of *Stellaria media* and renal cell carcinoma. (a) Venn diagram showing the overlap between *Stellaria media*‐related genes and renal cell carcinoma‐associated genes. (b) Interaction between active compounds and target genes involved in renal cell carcinoma.

**FIGURE 5:**
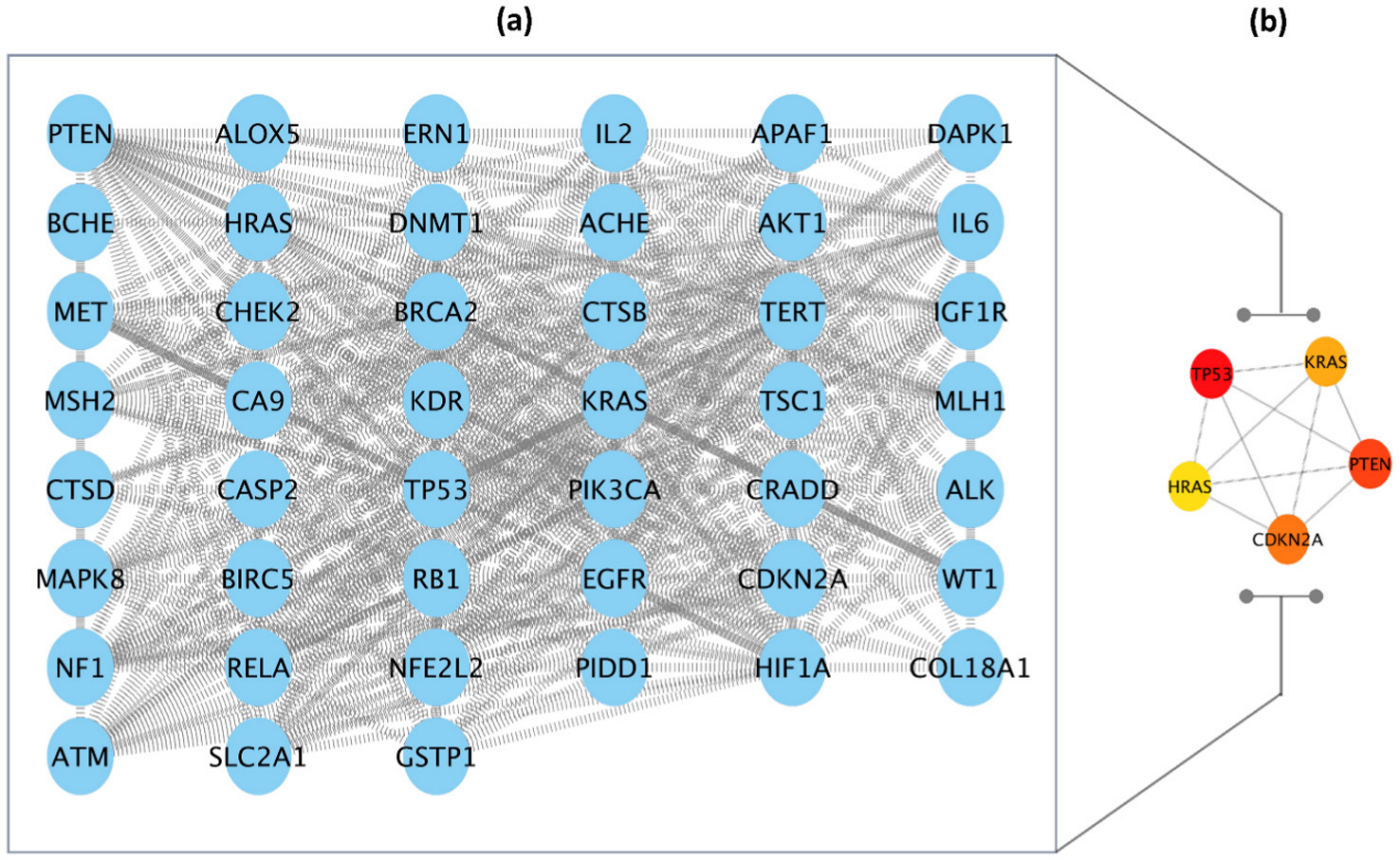
Renal cell carcinoma interaction network: (a) mapping the network of gene targets, (b) hub genes related renal cell carcinoma.

In order to identify genes associated with *S. media* phytochemicals, the SwissTarget, PubChem, and TCMSP databases were utilized. Following the elimination of duplicate gene pairs, a total of 434 target genes were identified. Among the compounds analyzed, naringenin, tricin, and eriodictyol were found to have the highest number of nodes. In contrast, apigenin 6‐C‐hexoside 8‐C‐pentoside was linked to a single target gene. The network map illustrates the common targets of these compounds, demonstrating the extensive many‐to‐many relationships between the compounds and their targets (Figure 4b). In order to identify genes associated with renal cell carcinoma, the CTD, String, GeneCards, and DisGeNET databases were referenced. In the case of the CTD database, only those genes with direct evidence were selected. A total of 45 genes were selected for further analysis (Figure 5a,b).

The analysis conducted using the STRING database involved these 45 cancer‐associated targets, resulting in the identification of a gene interaction network comprising 45 nodes and 405 edges. The network was visually represented by ellipses of varying sizes, with the size of each ellipse serving to highlight the relative importance of the gene within the network. In order to identify the key genes within this complex structure, the maximal clique centrality (MCC) method was employed via the CytoHubba plugin, which identified the following hub genes: The identified hub genes were TP53, CDKN2A, PTEN, KRAS, and HRAS (Figure 5a,b). The aforementioned hub genes were selected for docking studies, with the objective of further exploring their potential as therapeutic targets.

### 3.8 Molecular Docking

**FIGURE 6:**
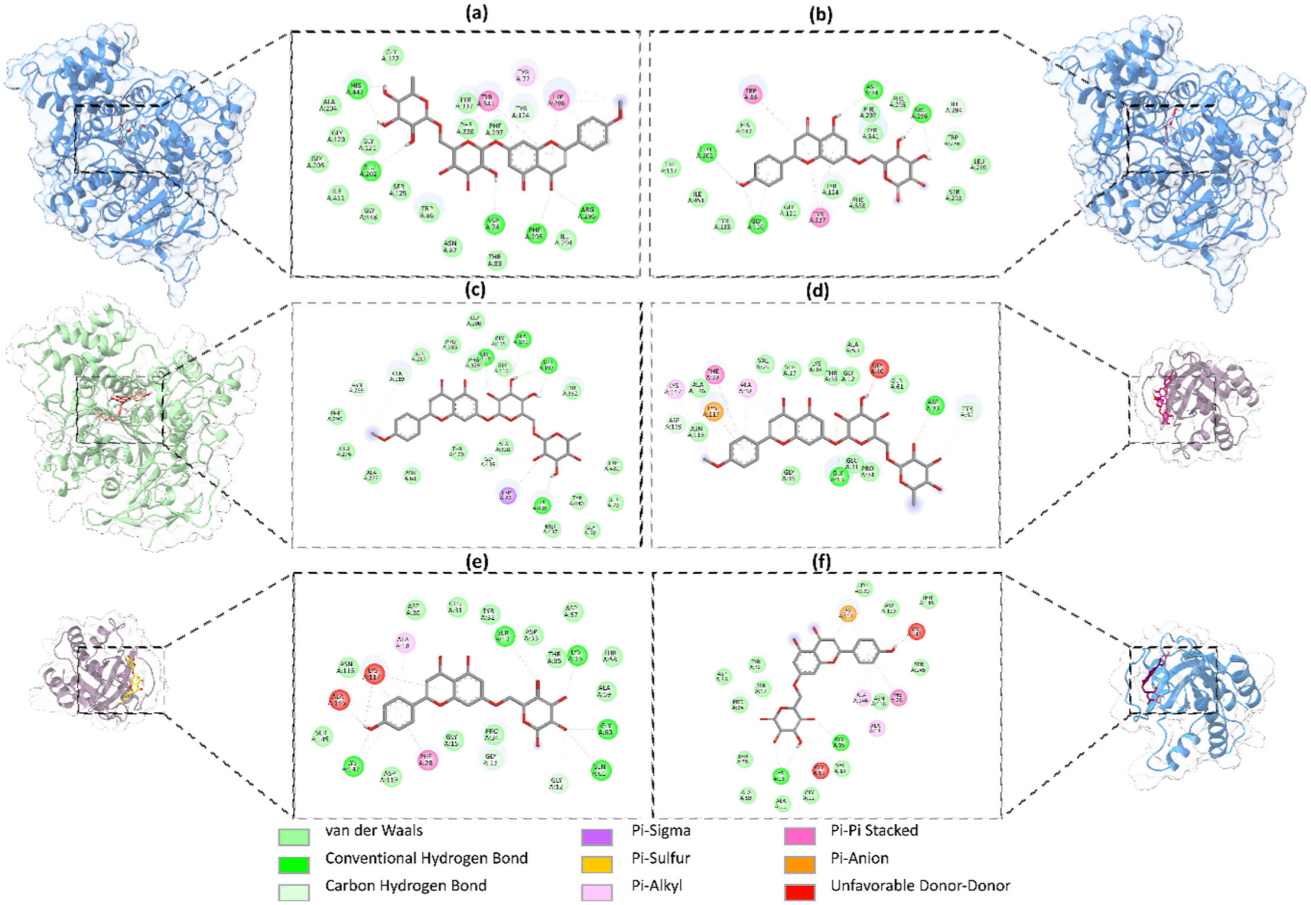
Enzymes’ and proteins' active sites with compounds showing the best binding energy: (a) Interaction between acacetin O‐hexoside‐O‐deoxyhexoside and AChE. (b) Interaction between napigenin 7‐O‐hexoside and AChE. (c) Interaction between acacetin O‐hexoside‐O‐deoxyhexoside and BChE. (d) Interaction between acacetin O‐hexoside‐O‐deoxyhexoside and HRAS. (e) Interaction between napigenin 7‐O‐hexoside and HRAS. (f) Interaction between napigenin 7‐O‐hexoside and KRAS.

As part of the study, molecular docking was conducted for enzymes and proteins. The requisite coordinates and grid sizes for these analyses are provided in Supplementary Table 1. From the multitude of compounds identified in *S. media*, aesculin, naringenin, acacetin O‐hexoside‐O‐deoxyhexoside, tricin, apigenin 6‐C‐hexoside 8‐C pentoside, napigenin‐7‐O‐hexoside, and eriodictyol were selected for comprehensive analysis due to their pervasive distribution. In the study of renal cell carcinoma, the selected proteins included TP53, CDKN2A, PTEN, KRAS, and HRAS, in addition to the enzymes AChE, BChE, Tyr, Amylase, and Glucosidase. The proteins were identified through a disease ontology enrichment analysis using the DOSE package, followed by the screening of disease‐associated genes in the STRING database. Figure 5a,b provides a visual representation of the genes commonly associated with renal cell carcinoma. The selected compounds exhibited binding energies ranging from −11.2 to −6.2 kcal/mol with both enzymatic targets and renal cell carcinoma‐associated proteins (Tables S1 and S2). Subsequently, compounds exhibiting a minimum docking score threshold of −8.0 kcal/mol and at least one hydrogen bond were selected for a more comprehensive evaluation. A hydrogen bond distance cutoff of 4 Å was applied in order to facilitate the evaluation process. Figure 6 provides a visual representation, and Table S2 lists the compounds that exceeded the docking score threshold of −8.0 kcal/mol.

The results of the molecular docking study show that out of seven molecules, two compounds, acacetin‐O‐hexoside‐O‐deoxyhexoside and napigenin 7‐O‐hexoside, have remarkable binding energies with proteins. Analysis of the binding energies for enzyme residues revealed higher interactions between AChE and acacetin O‐hexoside‐O‐deoxyhexoside, AChE and napigenin 7‐O‐hexoside, BChE and acacetin O‐hexoside‐O‐deoxyhexoside, respectively. In these interactions, it was observed that bonds such as pi‐pi, pi‐sigma, pi‐sulfur, pi‐alkyl, and conventional hydrogen bonds were more prevalent than hydrogen bonds (Figure 6). The detection of hydrogen bonds in the interactions between acacetin O‐hexoside‐O‐deoxyhexoside and AChE (Figure 6a), napigenin 7‐O‐hexoside and AChE (Figure 6b), acacetin O‐hexoside‐O‐deoxyhexoside and BChE (Figure 6c), acacetin O‐hexoside‐O‐deoxyhexoside and HRAS (Figure 6d), napigenin 7‐O‐hexoside and HRAS (Figure 6e), napigenin 7‐O‐hexoside and KRAS highlighted the significance of these interactions (Figure 6). The highest binding score observed was −10.1 kcal/mol (Table 6).

**TABLE 6:** The docking score (kcal/mol) and interacting residues of the enzyme and protein.

Compound	Receptor		Binding energy (kcal/mol)	RMSD	Interaction	Amino acid residues	
	Target	PDB ID			Type	Number	Binding site
Aesculin	Amylase	2qv4	−8.2	0.5198	H‐bond	2	Glu A:233
Naringenin	Amylase	2qv4	−8.9	0.1911	H‐bond	2	Gln A:63, Asp A:300
Acacetin‐O‐hexoside‐O‐deoxyhexoside	Amylase	2qv4	−9.6	2.6156	H‐bond	4	Asp A:300, His A:299, Glu A:233 (2)
Tricin	Amylase	2qv4	−8.1	0.9933	H‐bond	0	
Apigenin 6‐C‐hexoside 8‐C‐pentoside	Amylase	2qv4	−10.0	0.596	H‐bond	8	His A:305 (3), Glu A:233, Asp A:300, Gln A:63 (2), Thr A:163
Napigenin 7‐O‐hexoside	Amylase	2qv4	−9.5	7.6274	H‐bond	2	Glu A:233, His A:201
Eriodictyol	Amylase	2qv4	−9.1	1.0156	H‐bond	6	Gln A:63, Glu A:233, Asp A:197 (2), His A:299
Acacetin‐O‐hexoside‐O‐deoxyhexoside	Glucosidase	3w37	−8.7	0.9842	H‐bond	6	Arg A:552, Asp A:630, Asp A:232, Asp A:568 (3)
Apigenin 6‐C‐hexoside 8‐C‐pentoside	Glucosidase	3w37	−8.1	0.6559	H‐bond	2	Ser A:406, Thr A:409
Napigenin 7‐O‐hexoside	Glucosidase	3w37	−9.3	0.9854	H‐bond	2	Asp A:23, Asp A:357
Eriodictyol	Glucosidase	3w37	−8.2	0.1661	H‐bond	3	Asp A:357 (2), Asp A:232
Aesculin	AChE	2y2v	−9.3	0.8818	H‐bond	5	Trp A:86, Gly A:126, Ala A:204, Tyr A:341, Thr A:83
Naringenin	AChE	2y2v	−9.9	0.7023	H‐bond	4	Thr A:341 (2), Thr A:337, Thr A:124
Acacetin O‐hexoside‐O‐deoxyhexoside	AChE	2y2v	−11.5	0.2657	H‐bond	6	His A:447, Glu A:202 (2), Asp A:74, Phe A:295, Arg A:296
Tricin	AChE	2y2v	−9.5	0.6109	H‐bond	3	Thr A:337, Phe A:295, Ser A:293
Apigenin 6‐C‐hexoside 8‐C‐pentoside	AChE	2y2v	−9.8	0.5919	H‐bond	4	Gln A:291, Ser A:293, Arg A:296, Phe A:295
Napigenin 7‐O‐hexoside	AChE	2y2v	−11.1	4.653	H‐bond	4	Glu A:202, Gly A:120, Arg A:296, Asp A:274
Eriodictyol	AChE	2y2v	−9.9	0.5723	H‐bond	3	Thr A:341 (2), Thr A:124
Aesculin	BChE	3djy	−8.7	0.9659	H‐bond	4	Trp A:82, Trp A:440, Thr A:122, His A:438
Naringenin	BChE	3djy	−8.7	0.9843	H‐bond	2	Glu A:197, Gly A:115
Acacetin O‐hexoside‐O‐deoxyhexoside	BChE	3djy	−10.1	1.0193	H‐bond	5	Gly A:117, Glu A:197 (2), Ala A:199, His A:438
Tricin	BChE	3djy	−8.6	0.7419	H‐bond	1	Asp A:70
Apigenin 6‐C‐hexoside 8‐C‐pentoside	BChE	3djy	−9.7	0.6381	H‐bond	1	His A:438
Napigenin 7‐O‐hexoside	BChE	3djy	−10.0	0.7728	H‐bond	6	Glu A:197, Tyr A:128, Asn A:68, Asp A:70, His A:438, Tyr A:332
Eriodictyol	BChE	3djy	−9.1	1.0191	H‐bond	3	Asn A:68, Asp A:70, Asn A:83
Acacetin‐O‐hexoside‐O‐deoxyhexoside	Tyr	5m8o	−9.3	0.7353	H‐bond	10	Glu A:216, Arg A:374 (2), Tyr A:362 (2), Asn A:378 (2), Gln A:390, Asp A:212, Thr A:391
Napigenin‐7‐O‐hexoside	Tyr	5m8o	−9.5	0.7134	H‐bond	8	Arg A:374 (2), Ser A:394, Gly A:386, Asn A:385, Asn A:318 (2), Arg A:321
Acacetin‐O‐hexoside‐O‐deoxyhexoside	CDKN2A	1 dc2	−8.2	1.049	H‐bond	7	Asp A:108, Asp A:84, Arg A:46 (3), Tyr A:44, Tyr A:77
Aesculin	Hra‐S	1p2s	−8.5	1.0597	H‐bond	3	Asp A:33, Gly A:15, Glu A:31
Acacetin O‐hexoside‐O‐deoxyhexoside	Hra‐S	1p2s	−9.7	1.8465	H‐bond	3	Gly A:13 (2), Asp A:33
Tricin	Hra‐S	1p2s	−8.5	0.0684	H‐bond	2	Thr A:35, Gly A:13
Apigenin 6‐C‐hexoside 8‐C‐pentoside	Hra‐S	1p2s	−8.2	0.6451	H‐bond	6	Asp A:119, Lys A:147, Lys A:16, Thr A:35, Asp A:33, Lys A:117
Napigenin 7‐O‐hexoside	Hra‐S	1p2s	−11.2	0.515	H‐bond	5	Gln A:61, Gly A:60, Lys A:16, Ser A:17, Lys A:147
Eriodictyol	Hra‐S	1p2s	−8.7	2.1889	H‐bond	1	Glu A:31
Aesculin	Kra‐s	8afb	−8.0	1.0695	H‐bond	2	Thr A:32, Lys A:16
Naringenin	Kra‐s	8afb	−8.3	1.0607	H‐bond	2	Thr A:32, Ser A:145
Acacetin O‐hexoside‐O‐deoxyhexoside	Kra‐s	8afb	−9.1	0.7933	H‐bond	9	Lys A:147, Ser A:145, Val A:129, Tyr A: 32 (2), Glu A:31, Gly A:13 (2), Ser A:117
Tricin	Kra‐s	8afb	−9.0	0.3823	H‐bond	4	Tyr A:32, Ala A:18, Ser A:145, Asn A:116
Apigenin 6‐C‐hexoside 8‐C‐pentoside	Kra‐s	8afb	−8.8	0.5755	H‐bond	3	Tyr A:32, Val A:29, Lys A:147
Napigenin 7‐O‐hexoside	Kra‐s	8afb	−10.6	1.0536	H‐bond	4	Gly A:15 (2), Lys A:16 (2)
Eriodictyol	Kra‐s	8afb	−8.8	1.0295	H‐bond	3	Asp A:119, Ala A:146, Tyr A:32
Acacetin O‐hexoside‐O‐deoxyhexoside	Pten	1d5r	−9.5	0.1978	H‐bond	1	Lys A:332
Napigenin 7‐O‐hexoside	Pten	1d5r	−8.8	1.0482	H‐bond	7	Asp A:324, Asn A:323 (2), Gln A:149, Tyr A:176
Eriodictyol	Pten	1d5r	−8.1	0.8453	H‐bond	2	Asp A:324, Arg A:173
Acacetin O‐hexoside‐O‐deoxyhexoside	TP53	6mxy	−8.0	4.6987	H‐bond	1	Lys A:332

A molecular docking study was conducted to elucidate the inhibitory potential of various bioactive compounds against renal cell carcinoma. In this context, acacetin 7‐O‐hexoside‐O‐deoxyhexoside and napigenin 7‐O‐hexoside exhibited strong binding affinities with various target enzymes and proteins, thereby demonstrating their potential for therapeutic applications. With regard to amylase, apigenin 6‐C‐hexoside 8‐C‐pentoside exhibited the highest binding affinity (−10.0 kcal/mol), with the lower RMSD value of 0.596, interacting with key residues such as His A:305 (3), Glu A:233, and Asp A:300. Similarly, acacetin O‐hexoside‐O‐deoxyhexoside, with the highest RMSD value of 2.6156, and napigenin 7‐O‐hexoside, with the highest RMSD value of 7.6274, exhibited comparable interactions at the Glu A:233 residue, with binding affinities of −9.6 and −9.5 kcal/mol, respectively. In the case of BChE, acacetin O‐hexoside‐O‐deoxyhexoside, with a lower RMSD value of 1.0193, and napigenin 7‐O‐hexoside, with a lower RMSD value of 0.7728, exhibited robust and consistent binding interactions, particularly with the Glu A:197 and His A:438 residues. With regard to glucosidase, both compounds exhibited notable binding scores, with acacetin O‐hexoside‐O‐deoxyhexoside, with a lower RMSD value of 0.9842 (−8.7 kcal/mol), and napigenin 7‐O‐hexoside, with a lower RMSD value of 0.9854 (−9.3 kcal/mol), demonstrating significant interactions. With regard to AChE, acacetin O‐hexoside‐O‐deoxyhexoside, with a lower RMSD value of 0.2657 (−11.5 kcal/mol), and napigenin 7‐O‐hexoside, with the highest RMSD value of 4.653 (−11.1 kcal/mol), demonstrated interactions with residues such as Phe A:295, suggesting potential applications in the treatment of neurodegenerative diseases. On Tyr, acacetin O‐hexoside‐O‐deoxyhexoside, with a lower RMSD value of 0.7353 (−9.3 kcal/mol), and napigenin 7‐O‐hexoside, with a lower RMSD value of 0.7134 (−9.5 kcal/mol), demonstrated significant and consistent interactions with Arg A:374. In the context of HRAS interactions, napigenin 7‐O‐hexoside, with a lower RMSD value of 0.515 (−11.2 kcal/mol), and acacetin O‐hexoside‐O‐deoxyhexoside, with a lower RMSD value of 1.8465 (−9.7 kcal/mol), exhibited notable binding affinity, particularly with residues Gln A:61 and Lys A:16. With regard to KRAS, napigenin 7‐O‐hexoside, with a lower RMSD value of 1.0536 (−10.6 kcal/mol), and acacetin O‐hexoside‐O‐deoxyhexoside, with a lower RMSD value of 0.1978 value (−9.1 kcal/mol), exhibited the potential to act as effective inhibitors. In the case of Pten, acacetin O‐hexoside‐O‐deoxyhexoside, with a lower RMSD value of 0.1978 (−9.5 kcal/mol), and napigenin 7‐O‐hexoside, with a lower RMSD value of 1.0482 (−8.8 kcal/mol), demonstrated interactions. With regard to CDKN2A, acacetin O‐hexoside‐O‐deoxyhexoside, with a lower RMSD value of 1.049 (−8.2 kcal/mol), exhibited binding interactions with residues Asp A:108 and Arg A:46 (3). Finally, acacetin O‐hexoside‐O‐deoxyhexoside, with the highest RMSD value of 4.6987 (−8.0 kcal/mol), was observed to interact with Lys A:332 with regard to TP53. The results indicate that acacetin O‐hexoside‐O‐deoxyhexoside and naringenin 7‐O‐hexoside have the potential to serve as effective inhibitors in the treatment of renal cell carcinoma. These molecules demonstrate strong binding affinities to various enzymes and proteins, which suggests broad therapeutic potential.

### 3.9 Conclusion

In summary, the biological properties and chemical profiles of *S. media* extracts were extensively studied. Based on UHPLC–MS/MS results, the extracts were rich in flavonoids. In general, we did not observe any correlation between the tested biological properties. For example, although the ethyl acetate and ethanol extracts contained more phenolics compared to ethanol/water and water extracts, the extracts were the weakest in the DPPH assay. Interestingly, the water extract also showed the best scavenging ability in the ABTS assay. However, the ethyl acetate and ethanol extracts were more active in the enzyme inhibition tests. In terms of cell viability (%), the ethyl acetate, ethanol, and water extracts were as toxic to normal cell lines (HEK 293). Taken together, the differences can be explained by the complex nature of the phytochemicals and their interactions, for example, antagonistic or synergistic. Furthermore, the observed ability cannot be directly attributed to the presence of phenols and other compounds alone but can be attributed to the observed abilities. Network pharmacology and molecular docking analysis also revealed the interaction between the compounds (particularly flavonoids) and enzymatic targets as well as target genes. Our results suggest that *S. media* can be an alternative natural raw material instead of synthetic active ingredients in the pharmaceutical, nutraceutical, and cosmetic industries. However, further experimental studies strongly recommend isolating active ingredients and their effects as well as pharmacokinetic properties.

## Author Contributions

**Gaia Cusumano:** conceptualization (equal), data curation (equal), investigation (equal), writing – original draft (equal). **Giancarlo Angeles Flores:** conceptualization (equal), investigation (equal), methodology (equal), writing – original draft (equal). **Mehmet Veysi Cetiz:** conceptualization (equal), funding acquisition (equal), methodology (equal), visualization (equal). **Umran Kurt:** investigation (equal), validation (equal), writing – original draft (equal), writing – review and editing (equal). **Gunes Ak:** conceptualization (equal), investigation (equal), writing – original draft (equal). **Enver Saka:** investigation (equal), methodology (equal), writing – original draft (equal). **Shaza H. Aly:** data curation (equal), investigation (equal), writing – original draft (equal), writing – review and editing (equal). **Abdel Nasser Singab:** investigation (equal), methodology (equal), writing – review and editing (equal). **Gokhan Zengin:** conceptualization (equal), data curation (equal), investigation (equal), writing – original draft (equal), writing – review and editing (equal). **Ismail Senkardes:** resources (equal), validation (equal), writing – original draft (equal). **Maria J. Rodrigues:** investigation (equal), writing – original draft (equal), writing – review and editing (equal). **Carla Emiliani:** investigation (equal), methodology (equal), writing – original draft (equal). **Paola Angelini:** investigation (equal), methodology (equal), resources (equal). **Luisa Custodio:** conceptualization, methodology, writing – original draft. **Omayma A. Eldahshan:** conceptualization, methodology, writing – original draft.

## Conflicts of Interest

The authors declare no conflicts of interest.

## Supporting information


Table S1.

## Data Availability

The data that support the findings of this study are available on request from the corresponding author.

## References

[fsn34505-bib-0001] Abdelazim, E. B. , T. Abed , S. S. Goher , et al. 2024. “In Vitro and In Vivo Studies of Syzygium Cumini‐Loaded Electrospun PLGA/PMMA/Collagen Nanofibers for Accelerating Topical Wound Healing.” RSC Advances 14, no. 1: 101–117. 38173621 10.1039/d3ra06355k PMC10758764

[fsn34505-bib-0002] Abdelghffar, E. A. , H. A. El‐Nashar , A. G. Al‐Mohammadi , and O. A. Eldahshan . 2021. “Orange Fruit (Citrus Sinensis) Peel Extract Attenuates Chemotherapy‐Induced Toxicity in Male Rats.” Food & Function 12, no. 19: 9443–9455. 34606555 10.1039/d1fo01905h

[fsn34505-bib-0003] Abdelghffar, E. A. , H. A. El‐Nashar , S. Fayez , W. A. Obaid , and O. A. Eldahshan . 2022. “Ameliorative Effect of Oregano (Origanum Vulgare) Versus Silymarin in Experimentally Induced Hepatic Encephalopathy.” Scientific Reports 12, no. 1: 17854. 36284120 10.1038/s41598-022-20412-3 PMC9596437

[fsn34505-bib-0004] AbouZeid, E. M. , A. H. Afifi , A. Salama , et al. 2022. “Comprehensive Metabolite Profiling of Phoenix Rupicola Pulp and Seeds Using UPLC‐ESI‐MS/MS and Evaluation of Their Estrogenic Activity in Ovariectomized Rat Model.” Food Research International 157: 111308. 35761603 10.1016/j.foodres.2022.111308

[fsn34505-bib-0005] Ahmad, W. , M. Ahmad , M. U. K. Sahibzada , et al. 2022. “Lipid Peroxidation Reduction and Hippocampal and Cortical Neurons Protection Against Ischemic Damage in Animal Model Using Stellaria Media.” Saudi Journal of Biological Sciences 29, no. 3: 1887–1892. 35280571 10.1016/j.sjbs.2021.10.033 PMC8913427

[fsn34505-bib-0006] Aleem, A. , B. Aslam , M. B. Alim , A. Hussain , M. N. Faisal , and W. Majeed . 2023. “Phytochemical Analysis and Gastroprotective Effect of *Stellaria media* (L.) Vill. Methanolic Extract on Piroxicam‐Induced Gastric Ulcer in Wistar Rats.” Pakistan Journal of Pharmaceutical Sciences 36, no. 5: 1425–1434. 37869918

[fsn34505-bib-0007] Alqethami, A. , and A. Y. Aldhebiani . 2021. “Medicinal Plants Used in Jeddah, Saudi Arabia: Phytochemical Screening.” Saudi Journal of Biological Sciences 28, no. 1: 805–812. 33424370 10.1016/j.sjbs.2020.11.013 PMC7783804

[fsn34505-bib-0008] Aly, S. H. , O. A. Eldahshan , S. T. Al‐Rashood , et al. 2022. “Chemical Constituents, Antioxidant, and Enzyme Inhibitory Activities Supported by In‐Silico Study of n‐Hexane Extract and Essential Oil of Guava Leaves.” Molecules 27, no. 24: 8979. 36558111 10.3390/molecules27248979 PMC9781903

[fsn34505-bib-0009] Aly, S. H. , M. A. El‐Hassab , S. S. Elhady , and H. A. Gad . 2022. “Comparative Metabolic Study of Tamarindus Indica L.'s Various Organs Based on GC/MS Analysis, In Silico and In Vitro Anti‐Inflammatory and Wound Healing Activities.” Plants 12, no. 1: 87. 36616217 10.3390/plants12010087 PMC9824397

[fsn34505-bib-0010] Aly, S. H. , A. M. Elissawy , M. A. El Hassab , et al. 2024. “Comparative Metabolic Study of the Chloroform Fraction of Three Cystoseira Species Based on UPLC/ESI/MS Analysis and Biological Activities.” Journal of Enzyme Inhibition and Medicinal Chemistry 39, no. 1: 2292482. 38086785 10.1080/14756366.2023.2292482 PMC11721769

[fsn34505-bib-0011] Aly, S. H. , A. M. Elissawy , A. M. Fayez , O. A. Eldahshan , M. A. Elshanawany , and A. N. B. Singab . 2021. “Neuroprotective Effects of Sophora Secundiflora, Sophora Tomentosa Leaves and Formononetin on Scopolamine‐Induced Dementia.” Natural Product Research 35, no. 24: 5848–5852. 32696670 10.1080/14786419.2020.1795853

[fsn34505-bib-0013] Aly, S. H. , A. M. Elissawy , D. Salah , et al. 2023. “Phytochemical Investigation of Three Cystoseira Species and Their Larvicidal Activity Supported With In Silico Studies.” Marine Drugs 21, no. 2: 117. 36827158 10.3390/md21020117 PMC9967941

[fsn34505-bib-0014] Ancheeva, E. , G. Daletos , R. Muharini , W. H. Lin , L. Teslov , and P. Proksch . 2015. “Flavonoids From Stellaria Nemorum and Stellaria Holostea.” Natural Product Communications 10, no. 3: 1934578X1501000315. 25924523

[fsn34505-bib-0015] Arora, D. , and A. Sharma . 2012. “Evaluation of Anxiolytic Activity of Stellaria Media Linn. Extracts in Mice.” Pharmacology Communications 2: 58–61.

[fsn34505-bib-0016] Arora, D. , and A. Sharma . 2014. “Isolation and Characterization of the Chemical Constituents of Stellaria Media Linn.” International Journal of Pharmaceutical Sciences and Research 5, no. 9: 3669.

[fsn34505-bib-0017] Ayoub, I. M. , M. Korinek , M. El‐Shazly , et al. 2021. “Anti‐Allergic, Anti‐Inflammatory, and Anti‐Hyperglycemic Activity of Chasmanthe Aethiopica Leaf Extract and Its Profiling Using LC/MS and GLC/MS.” Plants 10, no. 6: 1118. 34073129 10.3390/plants10061118 PMC8226651

[fsn34505-bib-0018] Castillo‐Peinado, L. , M. López‐Bascón , A. Mena‐Bravo , M. L. de Castro , and F. Priego‐Capote . 2019. “Determination of Primary Fatty Acid Amides in Different Biological Fluids by LC–MS/MS in MRM Mode With Synthetic Deuterated Standards: Influence of Biofluid Matrix on Sample Preparation.” Talanta 193: 29–36. 30368294 10.1016/j.talanta.2018.09.088

[fsn34505-bib-0019] Castro‐Alvarez, A. , A. M. Costa , and J. Vilarrasa . 2017. “The Performance of Several Docking Programs at Reproducing Protein–Macrolide‐Like Crystal Structures.” Molecules 22, no. 1: 136. 28106755 10.3390/molecules22010136 PMC6155922

[fsn34505-bib-0020] Chon, S.‐U. , B.‐G. Heo , Y.‐S. Park , D.‐K. Kim , and S. Gorinstein . 2009. “Total Phenolics Level, Antioxidant Activities and Cytotoxicity of Young Sprouts of Some Traditional Korean Salad Plants.” Plant Foods for Human Nutrition 64: 25–31. 19016328 10.1007/s11130-008-0092-x

[fsn34505-bib-0021] Demján, V. , A. Sója , T. Kiss , et al. 2022. “Stellaria Media tea Protects Against Diabetes‐Induced Cardiac Dysfunction in Rats Without Affecting Glucose Tolerance.” Journal of Traditional and Complementary Medicine 12, no. 3: 250–259. 35493309 10.1016/j.jtcme.2021.08.003 PMC9039105

[fsn34505-bib-0022] Divito, E. B. , A. P. Davic , M. E. Johnson , and M. Cascio . 2012. “Electrospray Ionization and Collision Induced Dissociation Mass Spectrometry of Primary Fatty Acid Amides.” Analytical Chemistry 84, no. 5: 2388–2394. 22283789 10.1021/ac203158u

[fsn34505-bib-0023] Dominguez‐López, I. , M. Pérez , and R. M. Lamuela‐Raventós . 2023. “Total (Poly) Phenol Analysis by the Folin‐Ciocalteu Assay as an Anti‐Inflammatory Biomarker in Biological Samples.” Critical Reviews in Food Science and Nutrition 7: 1–7. 10.1080/10408398.2023.2220031 PMC10700652 37283051

[fsn34505-bib-0024] Duke, J. A. 2002. Handbook of Medicinal Herbs. New York, USA: CRC Press.

[fsn34505-bib-0025] Duran, T. , G. Peron , M. Zancato , et al. 2024. “Harnessing the Chemical Composition and Anti‐Oxidant, Anti‐Enzymatic, and Anti‐Cancer Activities of Two *Corydalis* Species (*C. erdelii* and C. *solida*) by Using In Vitro and In Silico Analysis.” Food Bioscience 61: 104762.

[fsn34505-bib-0026] Eldahshan, O. A. , N. A. Ayoub , A.‐N. B. Singab , and M. M. Al‐Azizi . 2009. “Potential Antioxidant Phenolic Metabolites From Doum Palm Leaves.” African Journal of Pharmacy and Pharmacology 3, no. 4: 158–164.

[fsn34505-bib-0027] Elebeedy, D. , A. Ghanem , S. H. Aly , et al. 2023. “Synergistic Antiviral Activity of Lactobacillus Acidophilus and Glycyrrhiza Glabra Against Herpes Simplex‐1 Virus (HSV‐1) and Vesicular Stomatitis Virus (VSV): Experimental and in Silico Insights.” BMC Microbiology 23, no. 1: 173. 37391715 10.1186/s12866-023-02911-z PMC10311774

[fsn34505-bib-0028] Elgindi, M. R. , A. E.‐N. B. Singab , S. H. Aly , and I. I. Mahmoud . 2016. “Phytochemical Investigation and Antioxidant Activity of Hyophorbe Verschaffeltii (Arecaceae).” Journal of Pharmacognosy and Phytochemistry 5, no. 2: 39–46.

[fsn34505-bib-0029] El‐Nashar, H. A. , S. H. Aly , A. Ahmadi , and M. El‐Shazly . 2022. “The Impact of Polyphenolics in the Management of Breast Cancer: Mechanistic Aspects and Recent Patents.” Recent Patents on Anti‐Cancer Drug Discovery 17, no. 4: 358–379. 34961465 10.2174/1574892816666211213090623

[fsn34505-bib-0030] El‐Nashar, H. A. , O. A. Eldahshan , N. F. A. Fattah , S. A. Loutfy , and I. M. Abdel‐Salam . 2023. “HPLC‐ESI/MS‐MS Characterization of Compounds in Dolomiaea Costus Extract and Evaluation of Cytotoxic and Antiviral Properties: Molecular Mechanisms Underlying Apoptosis‐Inducing Effect on Breast Cancer.” BMC Complementary Medicine and Therapies 23, no. 1: 354. 37803435 10.1186/s12906-023-04164-9 PMC10559653

[fsn34505-bib-0031] El‐Nashar, H. A. , W. M. Eldehna , S. T. Al‐Rashood , A. Alharbi , R. O. Eskandrani , and S. H. Aly . 2021. “GC/MS Analysis of Essential Oil and Enzyme Inhibitory Activities of Syzygium Cumini (Pamposia) Grown in Egypt: Chemical Characterization and Molecular Docking Studies.” Molecules 26, no. 22: 6984. 34834076 10.3390/molecules26226984 PMC8618078

[fsn34505-bib-0032] Fahmy, N. M. , S. Fayez , G. Zengin , et al. 2024. “Chemical Exploration of Different Extracts From Phytolacca Americana Leaves and Their Potential Utilization for Global Health Problems: ın Silico and Network Pharmacology Validation.” Journal of Biomolecular Structure and Dynamics 30: 1–21. 10.1080/07391102.2024.2308770 38288952

[fsn34505-bib-0033] Goher, S. S. , S. H. Aly , M. M. Abu‐Serie , et al. 2024. “Electrospun Tamarindus Indica‐Loaded Antimicrobial PMMA/Cellulose Acetate/PEO Nanofibrous Scaffolds for Accelerated Wound Healing: In‐Vitro and In‐Vivo Assessments.” International Journal of Biological Macromolecules 258: 128793. 38134993 10.1016/j.ijbiomac.2023.128793

[fsn34505-bib-0034] Grochowski, D. M. , S. Uysal , A. Aktumsek , et al. 2017. “In Vitro Enzyme Inhibitory Properties, Antioxidant Activities, and Phytochemical Profile of Potentilla Thuringiaca.” Phytochemistry Letters 20: 365–372.

[fsn34505-bib-0035] Güner, A. , and S. Aslan . 2012. Türkiye bitkileri listesi:(damarlı bitkiler). Istanbul, Turkey: Nezahat Gökyiǧit Botanik Bahçesi Yayınları.

[fsn34505-bib-0036] Hamdan, D. I. , S. Salah , W. H. B. Hassan , et al. 2022. “Anticancer and Neuroprotective Activities of Ethyl Acetate Fractions From Morus Macroura Miq. Plant Organs With Ultraperformance Liquid Chromatography‐Electrospray Ionization‐Tandem Mass Spectrometry Profiling.” ACS Omega 7, no. 18: 16013–16027. 35571826 10.1021/acsomega.2c01148 PMC9096986

[fsn34505-bib-0037] Hetmann, M. , C. Langner , V. Durmaz , et al. 2023. “Identification and Validation of Fusidic Acid and Flufenamic Acid as Inhibitors of SARS‐CoV‐2 Replication Using DrugSolver CavitomiX.” Scientific Reports 13, no. 1: 11783. 37479788 10.1038/s41598-023-39071-z PMC10362000

[fsn34505-bib-0038] Hodisan, V. , and A. Sancraian . 1989. “Triterpenoid Saponins From *Stellaria media* (L.) Cyr.” Farmácia 37: 105–109.

[fsn34505-bib-0039] Hussiny, S. , A. Elissawy , O. Eldahshan , M. Elshanawany , and A.‐N. Singab . 2020. “Phytochemical Investigation Using GC/MS Analysis and Evaluation of Antimicrobial and Cytotoxic Activities of the Lipoidal Matter of Leaves of Sophora Secundiflora and Sophora Tomentosa.” Archives of Pharmaceutical Sciences Ain Shams University 4, no. 2: 207–214.

[fsn34505-bib-0040] Katanić Stanković, J. S. , J. Đorović Jovanović , D. Mišić , et al. 2023. “UHPLC‐MS Phytochemical Profiling and Insight Into Bioactivity of Rabelera Holostea (Greater Stitchwort) Extract.” Molecules 28, no. 3: 1274. 36770939 10.3390/molecules28031274 PMC9921532

[fsn34505-bib-0041] Khan, R. , W. Ahmad , and M. Ahmed . 2019. “Stellaria Media Attenuates the Hyperglycemia and Hyperlipidemia in Alloxan‐Induced Diabetic Rat.” Bangladesh Journal of Pharmacology 14, no. 2: 80–86.

[fsn34505-bib-0042] Khedher, A. , S. Dhibi , H. Bouzenna , et al. 2022. “Antiulcerogenic and Antioxidant Activities of Plantago Ovata Ethanolic Extract in Rats.” Brazilian Journal of Biology 84: e255120. 10.1590/1519-6984.255120 35293532

[fsn34505-bib-0043] Kitanov, G. 1992. Phenolic acids and flavonoids from *Stellaria media* (L.) Vill.(Caryophyllaceae).

[fsn34505-bib-0044] Kripasana, K. , and J. Xavier . 2020. “Phytochemical Analysis and Antioxidant Activity of Leaf Extracts of Some Selected Plants of the Family Acanthaceae.” Plant Science Today 7, no. 2: 264–274.

[fsn34505-bib-0045] Kudumela, R. G. , T. E. Ramadwa , N. M. Mametja , and T. M. Masebe . 2024. “Corchorus Tridens L.: A Review of Its Botany, Phytochemistry, Nutritional Content and Pharmacological Properties.” Plants 13, no. 8: 1096. 38674505 10.3390/plants13081096 PMC11054996

[fsn34505-bib-0046] Li, J. , X. Yang , and S. Mehri . 2021. “Genetic Diversity in Stellaria L.(Caryophyllaceae) Using Sequence Related Amplified Polymorphism.” Genetika 53, no. 3: 1369–1377.

[fsn34505-bib-0047] Ma, L. , J. Song , Y. Shi , et al. 2012. “Anti‐Hepatitis B Virus Activity of Chickweed [*Stellaria media* (L.) Vill.] Extracts in HepG2. 2.15 Cells.” Molecules 17, no. 7: 8633–8646. 22810196 10.3390/molecules17078633 PMC6268626

[fsn34505-bib-0048] Miere, F. G. , M. Ganea , A. G. Teodorescu , et al. 2023. “Characterization in Terms of Phytochemical Content and Medicinal Potential of the Stellaria Media Plant Extract.” Pharmacophore 14, no. 1–2023: 45–55.

[fsn34505-bib-0049] Morita, H. , T. Kayashita , A. Shishido , K. Takeya , H. Itokawa , and M. Shiro . 1996. “Dichotomins A‐E, New Cyclic Peptides From *Stellaria dichotoma* L. Var. lanceolata Bge.” Tetrahedron 52, no. 4: 1165–1176.

[fsn34505-bib-0050] Narvarte, B. C. V. , T. G. T. Genovia , L. A. R. Hinaloc , et al. 2023. “Total Polyphenol Content of Tropical Marine and Coastal Flora: Potentials for Food and Nutraceutical Applications.” Journal of Applied Phycology 35, no. 5: 2431–2443.

[fsn34505-bib-0051] Nilofar, N. , G. Zengin , M. Acar , et al. 2024. “Assessing the Chemical Composition, Antioxidant and Enzyme Inhibitory Effects of Pentapleura Subulifera and Cyclotrichium Glabrescens Extracts.” Chemistry & Biodiversity 21, no. 2: e202301651. 38016080 10.1002/cbdv.202301651

[fsn34505-bib-0052] Oladeji, O. S. , and A. K. Oyebamiji . 2020. “Stellaria Media (L.) Vill.‐A Plant With Immense Therapeutic Potentials: Phytochemistry and Pharmacology.” Heliyon 6, no. 6: e04150. 32548330 10.1016/j.heliyon.2020.e04150 PMC7284062

[fsn34505-bib-0053] Rakhimzhanova, A. , Ö. Kılınçarslan , and R. Mammadov . 2018. “ *Stellaria media* ekstraktlarının antioksidan aktivitesinin belirlenmesi ve fenolik bileşenlerinin karakterizasyonu.” Ordu Üniversitesi Bilim Ve Teknoloji Dergisi 8, no. 2: 165–173.

[fsn34505-bib-0054] Raslan, M. A. , R. F. Taher , A. A. Al‐Karmalawy , et al. 2021. “ *Cordyline fruticosa* (L.) A. Chev. Leaves: Isolation, HPLC/MS Profiling and Evaluation of Nephroprotective and Hepatoprotective Activities Supported by Molecular Docking.” New Journal of Chemistry 45, no. 47: 22216–22233.

[fsn34505-bib-0055] Rodrigues, M. J. , V. Neves , A. Martins , et al. 2016. “In Vitro Antioxidant and Anti‐Inflammatory Properties of Limonium Algarvense flowers' Infusions and Decoctions: A Comparison With Green Tea (Camellia Sinensis).” Food Chemistry 200: 322–329. 26830595 10.1016/j.foodchem.2016.01.048

[fsn34505-bib-0056] Saber, F. R. , P. E. Munekata , K. Rizwan , et al. 2024. “Family Myrtaceae: The Treasure Hidden in the Complex/Diverse Composition.” Critical Reviews in Food Science and Nutrition 64, no. 19: 6737–6755. 36748791 10.1080/10408398.2023.2173720

[fsn34505-bib-0057] Salinitro, M. , A. van der Ent , A. Tognacchini , and A. Tassoni . 2020. “Stress Responses and Nickel and Zinc Accumulation in Different Accessions of Stellaria Media (L.) Vill. In Response to Solution pH Variation in Hydroponic Culture.” Plant Physiology and Biochemistry 148: 133–141. 31958680 10.1016/j.plaphy.2020.01.012

[fsn34505-bib-0058] Saracila, M. , T. D. Panaite , C. P. Papuc , and R. D. Criste . 2021. “Heat Stress in Broiler Chickens and the Effect of Dietary Polyphenols, With Special Reference to Willow (Salix Spp.) Bark Supplements—A Review.” Antioxidants 10, no. 5: 686. 33925609 10.3390/antiox10050686 PMC8146860

[fsn34505-bib-0059] Shan, Y. , J. Zhou , H. Guang Zhao , X. Feng , Y. Dong , and B. Xia . 2010. “Amino‐Acid and Mineral Composition of Stellaria Media.” Chemistry of Natural Compounds 46: 667–668.

[fsn34505-bib-0060] Shinde, N. V. , M. Himaja , S. Bhosale , M. Ramana , and D. Sakarkar . 2008. “Synthesis and Biological Evaluation of Delavayin‐C.” Indian Journal of Pharmaceutical Sciences 70, no. 6: 827–831. 21369456 10.4103/0250-474X.49137 PMC3040889

[fsn34505-bib-0061] Singab, A. , D. Bahgat , E. Al‐Sayed , and O. Eldahshan . 2015. “Saponins From Genus Albizia: Phytochemical and Biological Review.” Medicinal and Aromatic Plants 3, no. 1: 1–7.

[fsn34505-bib-0062] Singab, A. N. B. , N. M. Mostafa , O. A. Eldahshan , M. L. Ashour , and M. Wink . 2014. “Profile of Volatile Components of Hydrodistilled and Extracted Leaves of Jacaranda Acutifolia and Their Antimicrobial Activity Against Foodborne Pathogens.” Natural Product Communications 9, no. 7: 1934578X1400900731. 25230515

[fsn34505-bib-0063] Subramoniam, A. 2014. “Present Scenario, Challenges and Future Perspectives in Plant Based Medicine Development.” Annals of Phytomedicine 3, no. 1: 31–36.

[fsn34505-bib-0064] Sun, Y. , X. Zhang , X. Xue , Y. Zhang , H. Xiao , and X. Liang . 2009. “Rapid Identification of Polyphenol C‐Glycosides From Swertia Franchetiana by HPLC—ESI‐MS—MS.” Journal of Chromatographic Science 47, no. 3: 190–196. 19298704 10.1093/chromsci/47.3.190

[fsn34505-bib-0065] Syed Salleh, S. N. A. , N. A. Mohd Hanapiah , H. Ahmad , W. L. Wan Johari , N. H. Osman , and M. R. Mamat . 2021. “Determination of Total Phenolics, Flavonoids, and Antioxidant Activity and GC‐MS Analysis of Malaysian Stingless Bee Propolis Water Extracts.” Scientifica 2021, no. 1: 3789351. 34721923 10.1155/2021/3789351 PMC8556095

[fsn34505-bib-0066] Tan, J. N. , S. Mohd Saffian , F. Buang , et al. 2020. “Antioxidant and Anti‐Inflammatory Effects of Genus Gynura: A Systematic Review.” Frontiers in Pharmacology 11: 504624. 33328981 10.3389/fphar.2020.504624 PMC7734347

[fsn34505-bib-0200] Trott, O. , and A. J. Olson . 2010. “AutoDock Vina: Improving the Speed and Accuracy of Docking With a New Scoring Function, Efficient Optimization, and Multithreading.” Journal of Computational Chemistry 31, no. 2: 455–461. 19499576 10.1002/jcc.21334 PMC3041641

[fsn34505-bib-0067] Tutenocakli, T. 2023. “Plants Consumed As Food In Ethnobotanical Perspective: The Case Study Of YENICE‐CANAKKALE‐Turkiye.” Current Trends in Natural Sciences 12, no. 23: 224–233.

[fsn34505-bib-0068] Veeresham, C. 2012. “Natural Products Derived From Plants as a Source of Drugs.” Journal of Advanced Pharmaceutical Technology & Research 3, no. 4: 200–201. 23378939 10.4103/2231-4040.104709 PMC3560124

[fsn34505-bib-0069] Yagi, S. , G. Zengin , O. A. Eldahshan , et al. 2024. “Functional Constituents of Colchicum Lingulatum Boiss. & Spruner Subsp. Rigescens K. Perss. Extracts and Their Biological Activities With Different Perspectives.” Food Bioscience 60: 104496.

[fsn34505-bib-0070] Yu, G. , L.‐G. Wang , G.‐R. Yan , and Q.‐Y. He . 2014. “DOSE: An R/Bioconductor Package for Disease Ontology Semantic and Enrichment Analysis.” Bioinformatics 31, no. 4: 608–609. 25677125 10.1093/bioinformatics/btu684

[fsn34505-bib-0100] Zengin, G. , and A. Aktumsek . 2014. “Investigation of Antioxidant Potentials of Solvent Extracts From Different Anatomical Parts of Asphodeline Anatolica E. Tuzlaci: An Endemic Plant to Turkey.” African Journal of Traditional, Complementary and Alternative Medicines 11, no. 2: 481–488. 10.4314/ajtcam.v11i2.37 PMC4202661 25435637

[fsn34505-bib-0071] Zengin, G. , N. M. Fahmy , K. I. Sinan , et al. 2022. “Differential Metabolomic Fingerprinting of the Crude Extracts of Three Asteraceae Species With Assessment of Their In Vitro Antioxidant and Enzyme‐Inhibitory Activities Supported by In Silico Investigations.” PRO 10, no. 10: 1911.

[fsn34505-bib-0072] Zengin, G. , S. Yagi , O. A. Eldahshan , et al. 2024. “Decoding Chemical Profiles and Biological Activities of Aerial Parts and Roots of Eryngium Thorifolium Boiss by HPLC‐MS/MS, GC‐MS and In Vitro Chemical Assays.” Food Bioscience 61: 104556.

