# Supplementary material for: Small Steps to the Big Picture for Health‐Promoting Applications Through the Use of Chickweed (Stellaria media): In Vitro, In Silico, and Pharmacological Network Approaches
Source: Food Sci Nutr. 2024 Oct 3;12(11):9295–313. doi: 10.1002/fsn3.4505 (PMC11606822; doi:10.1002/fsn3.4505)
Supplement: Supplementary file 1 — Table S1. [file FSN3-12-9295-s001.docx]

**Small steps to the big picture for health-promoting applications through the use of chickweed (*Stellaria media*): *In vitro, in silico* and pharmacological network approaches.**

Gaia Cusumano^1^, Giancarlo Angeles Flores^1,2^, Mehmet Veysi Cetiz^3,4^, Ümran Kurt^5^, Gunes Ak^4^, Enver Saka^4^, Shaza H. Aly^6^, Omayma A. Eldahshan^7,8^, Abdel Nasser Singab^7,8^, Gokhan Zengin^4*^, Ismail Senkardes^9^, Maria J. Rodrigues^10^, Luisa Custodio^10^, Carla Emiliani^1^, Paola Angelini^1^,

*^1^Department of Chemistry, Biology and Biotechnology, University of Perugia, Via del Giochetto,06123 Perugia, Italy*

*^2^Botanic Garden “Giardino dei Semplici”, Department of Pharmacy, “Gabriele d’Annunzio” University,66100 Chieti, Italy*

*^3^Department of Bioinformatics, Biocenter, University of Wurzburg, Am Hubland 97074, Wurzburg, Germany*

*^4^Department of Biology, Science Faculty, Selcuk University, Konya, Turkey*

*^5^Recep Tayyip Erdogan University, Department of Chemistry, 53100, Rize, Turkiye*

*^6^Department of Pharmacognosy, Faculty of Pharmacy, Badr University in Cairo (BUC), Cairo, 11829, Egypt*

*^7^Department of Pharmacognosy, Faculty of Pharmacy, Ain Shams University, 11566, Cairo, Egypt*

*^8^Center for Drug Discovery Research and Development, Ain Shams University, Cairo, 11566, Egypt*

*^9^Department of Pharmaceutical Botany, Pharmacy Faculty, Marmara University, Istanbul, Turkey.*

*^10^Centre of Marine Sciences, University of Algarve, Campus of Gambelas, 8005-139 Faro, Portugal*

^*^Corresponding author: gokhanzengin@selcuk.edu.tr

**Supplementary Tables**

**Table S1.** Relevant protein and enzyme target coordinates of the docking box.

| **Receptor** | | **Coordinate** | | **Reference** |
| --- | --- | --- | --- | --- |
| **Target** | **PDB ID** | **Grid size X, Y, Z** | **X, Y, Z dimensions** |  |
| AChE | 2y2v | 22 Å X 30 Å X 40 Å | 31.062, 20.311, 11.947 | (Yagi et al., 2024} |
| BChE | 3djy | 30 Å X 30 Å X 30 Å | 44.794, -19.63, -25.227 | (Yagi et al., 2024) |
| Tyr | 5m8o | 26 Å X 26 Å X 28 Å | -13.194, 5.341, -26.28 | (Yagi et al., 2024 |
| Amylase | 2qv4 | 28 Å X 28 Å X 24 Å | 14.188, 48.964, 22.886 | (Yagi et al., 2024) |
| Glucosidase | 3w37 | 42 Å X 52 Å X 54 Å | 3.091, −8.008, −4.08 | (Patil et al., 2022) |
| TP53 | 6mxy | 28 Å X 40 Å X24 Å | -11.077, 23.211, -6.103 | (Praveen, 2024 #91 |
| CDKN2A | 1dc2 | 48 Å X 104 Å X 62 Å | -2.003, -0.127, -17.287 | (Hande et al., 2021) |
| KRAS | 8afb | 25 Å X 25 Å X 25 Å | 18.479, −8.144, 22.453 | (Alarmi et al., 2023) |
| HRAS | 1p2s | 50 Å X 30 Å X 36 Å | 8.731, 35.93, 19.722 | (Buhrman et al., 2003) |
| PTEN | 1d5r | 88 Å X 54 Å X 124 | 37.029, 78.446, 31.742 | (Mondal et al., 2020) |

**Table S2** Relevant protein and enzyme result of the docking scores

| **Compound and Receptor**  **Receptor** | | | | **Binding energy** | | **Receptor** | | **Binding energy** | **Receptor** | | **Binding energy** | | **Receptor** | **Binding energy** | | **Receptor** | **Binding energy** |
| --- | --- | --- | --- | --- | --- | --- | --- | --- | --- | --- | --- | --- | --- | --- | --- | --- | --- |
| **Compound** | | **PDB ID** | |  |  | **PDB ID** | |  | **PDB ID** | |  |  | **PDB ID** |  |  | **PDB ID** |  |
| **Enzyme** | aesculin | | 2qv4 | -8.2 | 3w37 | | -7.9 | | 2y2v | -9.3 | | 3djy | | -8.7 | 5m8o | | -7.5 |
|  | naringenin | | 2qv4 | -8.9 | 3w37 | | -7.9 | | 2y2v | -9.9 | | 3djy | | -8.7 | 5m8o | | -7.8 |
|  | acacetin O-hexoside-O-deoxyhexoside | | 2qv4 | -9.6 | 3w37 | | -8.7 | | 2y2v | -11.5 | | 3djy | | -10.1 | 5m8o | | -9.3 |
|  | tricin | | 2qv4 | -8.1 | 3w37 | | -7.8 | | 2y2v | -9.5 | | 3djy | | -8.6 | 5m8o | | -7.0 |
|  | apigenin 6-C-hexoside  8-C-pentoside | | 2qv4 | -10.0 | 3w37 | | -8.1 | | 2y2v | -9.8 | | 3djy | | -9.7 | 5m8o | | -7.3 |
|  | napigenin 7-O-hexoside | | 2qv4 | -9.5 | 3w37 | | -9.3 | | 2y2v | -11.1 | | 3djy | | -10.0 | 5m8o | | -9.5 |
|  | eriodictyol | | 2qv4 | -9.1 | 3w37 | | -8.2 | | 2y2v | -9.9 | | 3djy | | -9.1 | 5m8o | | -7.5 |
| **Protein** | aesculin | | 1dc2 | -6.6 | 1p2s | | -8.5 | | 8afb | -8.0 | | 1d5r | | -7.4 | 6mxy | | -7.4 |
|  | naringenin | | 1dc2 | -7.0 | 1p2s | | -8.5 | | 8afb | -8.3 | | 1d5r | | -7.8 | 6mxy | | -6.8 |
|  | acacetin O-hexoside-O-deoxyhexoside | | 1dc2 | -8.2 | 1p2s | | -9.7 | | 8afb | -9.1 | | 1d5r | | -9.5 | 6mxy | | -8.0 |
|  | tricin | | 1dc2 | -6.2 | 1p2s | | -8.5 | | 8afb | -9.0 | | 1d5r | | -7.3 | 6mxy | | -6.8 |
|  | apigenin 6-C-hexoside  8-C-pentoside | | 1dc2 | -6.9 | 1p2s | | -8.2 | | 8afb | -8.8 | | 1d5r | | -7.9 | 6mxy | | -7.8 |
|  | napigenin-7-O-hexoside | | 1dc2 | -7.8 | 1p2s | | -11.2 | | 8afb | -10.6 | | 1d5r | | -8.8 | 6mxy | | -7.8 |
|  | eriodictyol | | 1dc2 | -7.1 | 1p2s | | -8.7 | | 8afb | -8.8 | | 1d5r | | -8.1 | 6mxy | | -6.7 |
